# Structure and photophysical properties of copper(I) photosensitizers: a computational study of substituent effects

**DOI:** 10.1007/s00894-026-07025-7

**Published:** 2026-11-07

**Authors:** Bruno Campana Severino, Vania Martins Ramos, Ana Paula de Lima Batista

**Affiliations:** Departamento de Química, Grupo Computacional de Catálise e Espectroscopia (GCCE), Universidade Federal de São Carlos (UFSCar), Rod. Washington Luís, São Carlos, 13565-905 SP Brazil

**Keywords:** Cu(I) photosensitizers, Electronic excited states, Quantum chemical calculations

## Abstract

**Context:**

Developing efficient and sustainable photosensitizers is crucial for advancing solar energy conversion and light-driven chemical processes. Ruthenium(II) and iridium(III) complexes have traditionally dominated this field due to their favorable photophysical properties, but their high cost has prompted the search for alternatives using earth-abundant metals. Copper(I) complexes are promising because they are cost-effective, have a tunable electronic structure, and feature a $d^{10}$ configuration that avoids low-lying metal-centered excited states. However, further improvements are needed to match the performance of Ru(II) and Ir(III) complexes, with ligand engineering playing a key role. Although there has been progress, the systematic effect of R substituents on the N$^\wedge$N^R^ ligand in heteroleptic Cu(N$^\wedge$N^R^)(NacNac) complexes remains underexplored.

**Methods:**

We investigated a series of Cu(I) complexes of the type Cu(N$^\wedge$N^R^) (NacNac^Me^), where N$^\wedge$N is 2,2′-biquinoline and NacNac is β-diketiminate, using electronic structure calculations. The N$^\wedge$N^R^ ligand was systematically substituted with R groups including -NH$_2$, -OH, -CH$_3$, -H, -Cl, -COOCH$_3$, and -CF$_3$ to examine how these modifications affect the structure and photophysical properties of copper(I) photosensitizers. Ground- and excited-state properties were evaluated using density functional theory (DFT) and time-dependent DFT (TD-DFT), with excited-state character quantified using the TheoDORE program. We analyzed structural parameters, frontier orbital energies, and electronic transitions to correlate substituent electronic character with photophysical behavior. Local energy decomposition (LED) analyses based on DLPNO-CCSD(T) calculations were also performed to study metal-ligand interactions. The results indicate that strong electron-withdrawing groups such as -COOCH$_3$ and -CF$_3$ reduce the HOMO–LUMO gap and cause red shifts in charge-transfer absorption. These findings establish how substituent electronics modulate the frontier-orbital energies and charge-transfer absorption of this series of heteroleptic Cu(I) complexes, providing an electronic-structure framework for tuning their optical response.

**Supplementary Information:**

The online version contains supplementary material available at https://doi.org/10.1007/s00894-026-07025-7.

## Introduction

The global demand for sustainable energy solutions is increasing as efforts to mitigate climate change and decrease dependence on fossil fuels [1–3]. Solar energy conversion technologies are at the forefront of this transition, providing clean and renewable power generation [4, 5]. Photosensitizers are central to many of these technologies; these systems harvest light and transfer charge or energy to target species [6–8]. These compounds are essential in various light-induced processes and have demonstrated successful applications in dye-sensitized solar cells, photodynamic therapy, and the activation of photochemical reactions [9–15].

**Fig. 1:**
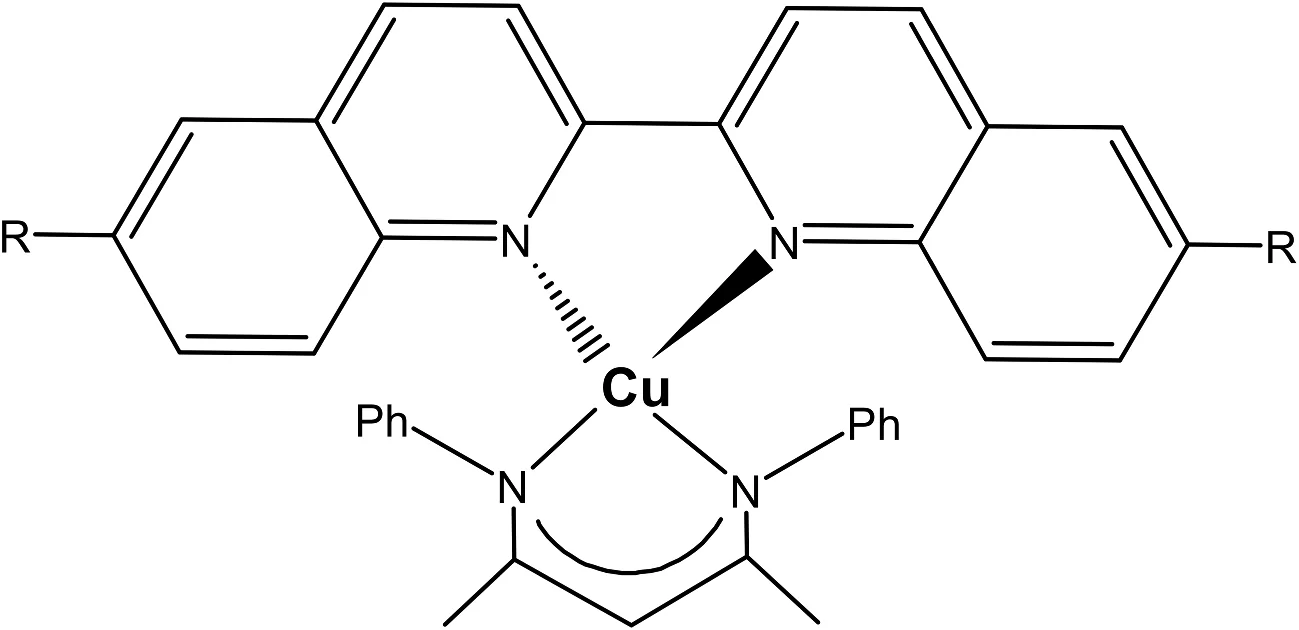
Complexes investigated in this work: Cu(N$^\wedge$N^R^)(NacNac^Me^) with R= -NH$_2$, -OH, -CH$_3$, -H, -Cl, -COOCH$_3$, and -CF$_3$. Me is the CH$_3$ group

Among the photosensitizers, noble-metal complexes, particularly those based in ruthenium(II) and iridium(III), have long dominated the field due to their broad absorption profiles, favorable redox potentials, and long-lived excited states [16–26]. However, the high cost and scarcity of these metals impose significant economic and environmental limitations, motivating the search for earth-abundant alternatives capable of achieving comparable photophysical performance [27–30].

In this context, copper(I) complexes have emerged as promising candidates: they combine earth abundance and low cost with a $d^{10}$ configuration which avoids non-radiative relaxation of metal-to-ligand charge transfer (MLCT) states via low-lying metal-centered (MC) states, a major issue in many first-row transition-metal photosensitizers [31]. For instance, Cu(I) bis(diimine) complexes have stand out, but they need improvements to prevent pseudo-Jahn-Teller (PJT) distortions, a pathway that destabilizes the target MLCT state [28, 32–37]. Upon visible-light excitation, oxidation to Cu(II) triggers a geometric flattening from an initially pseudotetrahedral environment toward a more planar configuration, lowering symmetry and exposing a fifth coordination site. The resulting transient Cu(II) species readily forms exciplexes with Lewis-basic solvents, leading to rapid excited-state quenching [28, 38–41].

Considerable progress has been made in mitigating this deactivation pathway through steric design: bulky substituents at the 2,9- and 3,8-positions of phenanthroline ligands restrict flattening, markedly extending MLCT lifetimes and enhancing photoluminescence [42–50]. Substituent electronics also play a crucial role, with electron-donating alkyl groups shifting redox potentials to more negative values and electron-withdrawing groups such as -CF$_3$ exerting the opposite inductive effect. Strongly withdrawing substituents at these positions have shown a profound influence on Cu(I) photophysics, demonstrating the power of targeted ligand modification to tune excited-state properties [50, 51].

Ligand engineering is central to optimizing Cu(I)-based photosensitizers. Classical N$^\wedge$N diimine ligands, such as biquinolines, offer extended π-conjugation that allows precise tuning of light absorption. Complementarily, β-diketimi-nate (NacNac) ligands offer strong σ-donation, steric protection of the metal center, and electronic flexibility [52–55]. In heteroleptic Cu(N$^\wedge$N)(NacNac^Me^) systems (Fig. 1), combining these ligand families enables dual-site modulation of the HOMO–LUMO gap: NacNac substitutions primarily affect the HOMO, while changes in the N$^\wedge$N ligand influence the LUMO. Furthermore, recent studies [54, 55] have demonstrated that more electron-rich NacNac ligands destabilize the HOMO. In contrast, π-extended N$^\wedge$N ligands (e.g., biquinoline) can significantly lower the LUMO energy. These effects jointly red-shift the MLCT/LLCT absorption band deep into the visible or near-infrared region [54–56].

The Cu(N$^\wedge$N^H^)(NacNac^Me^) system, for example, reached a charge-transfer absorption maximum around 800 nm, substantially beyond the blue/green absorption typical of copper bis-diimine systems. Despite this promising shift, the complex did not exhibit detectable emission in solution, and attempts to photocatalyze have so far been unsuccessful, strongly suggesting a short excited-state lifetime. In response, current efforts focus on steric tuning of both ligand frameworks to reduce excited-state distortion and overcome these limitations. The goal is to ultimately exploit their intense, long-wavelength visible absorption in photocatalytic applications [55].

An important but underexplored aspect of ligand tuning in Cu(I) photosensitizers is the systematic variation of the R-groups in N$^\wedge$N^R^ ligands. Although the qualitative influence of electron-donating and electron-withdrawing substituents on the electronic structure and MLCT transitions of Cu(I) complexes has been recognized [54–56], a detailed understanding of how these substituents influence molecular structure, frontier orbital energetics, metal–ligand interactions, and charge-transfer transitions within a single family of complexes remains limited. Here, we investigate a series of heteroleptic Cu(N$^\wedge$N^R^)(NacNac^Me^) complexes in which only the electronic nature of the R substituent is varied, allowing its influence to be directly assessed. Specifically, R spans electron-donating (-NH$_2$, -OH, -CH$_3$) to electron-withdrawing (-Cl, -COOCH$_3$, -CF$_3$) groups, with -H serving as a reference, as shown in Fig. 1. Density functional theory (DFT and TD-DFT) calculations, combined with excited-state character analysis performed with the TheoDORE program through quantitative charge-transfer descriptors, were employed to examine the relationships between substituent electronic character, molecular structure, frontier orbital energetics, and charge-transfer excitations. In addition, local energy decomposition (LED) analysis based on DLPNO-CCSD(T) calculations was used to investigate how substituent electronics modify individual metal–ligand interaction components. Together, these approaches provide new insights into the role of ligand substitution in heteroleptic Cu(I) photosensitizers.

## Computational details

This section is organized as follows:

i) Selection of an appropriate density functional and LED analysis:

**Fig. 2:**
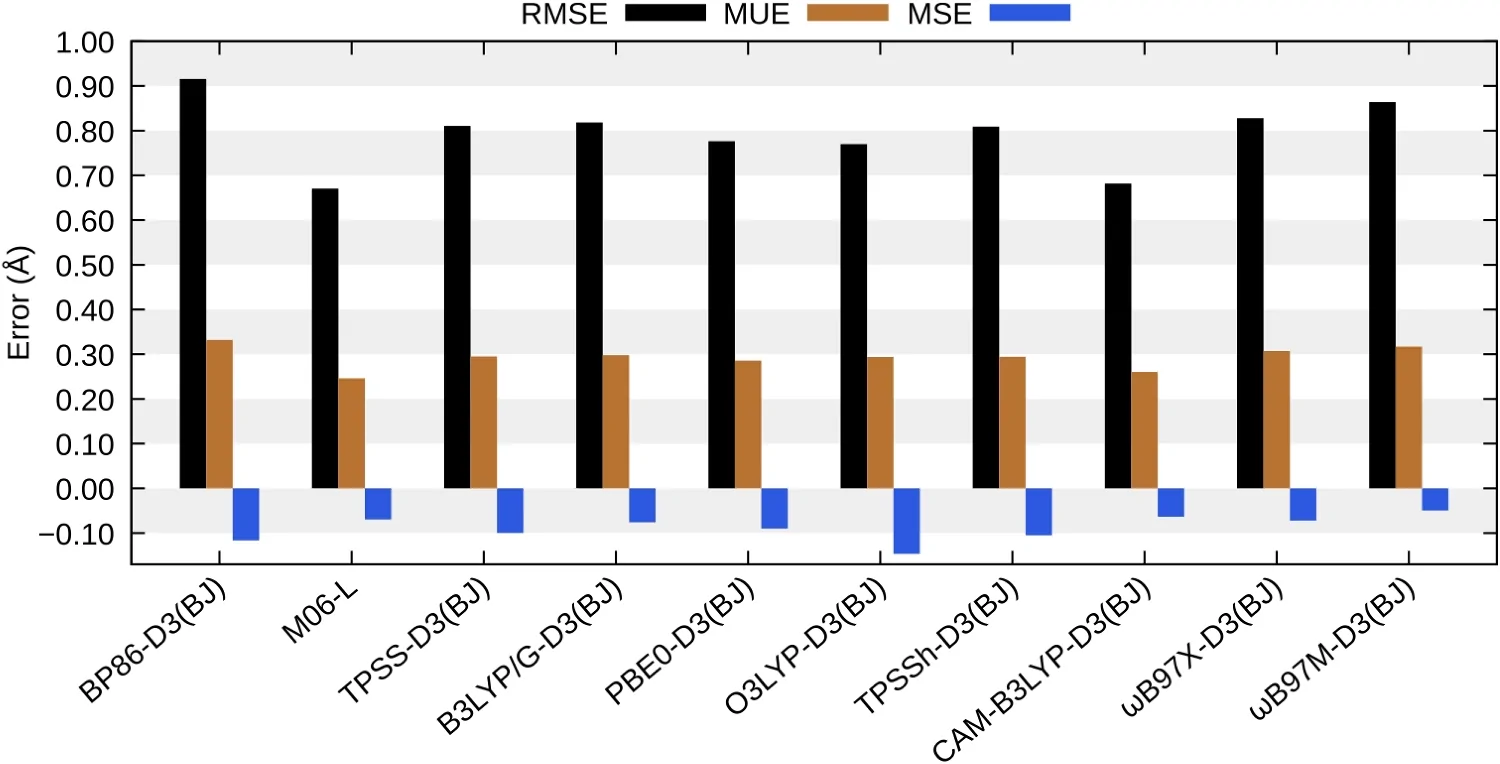
Analysis of RMSE, MUE, and MSE, comparing the X-ray structure of Cu(N$^\wedge$N^H^)(NacNac^Me^) with the corresponding DFT-optimized geometry obtained using different density functionals

The DFT selection is based on the experimental geometry reported for the Cu(N$^\wedge$N^H^)(NacNac^Me^) complex [54], here referred to as the parent Cu(I) complex. From this selection, a series of Cu(I) analogues were studied, all in their singlet ground electronic state, in which different R substituents are introduced in the N$^\wedge$N^R^ (2,2′-biquinoline) ligand: R= -NH$_2$, -OH, -CH$_3$, -Cl, -COOCH$_3$ and -CF$_3$. All these copper complexes exhibit charge 0. The focus was on the variations in the N$^\wedge$N region only, because modifications on the NacNac^Me^ (β-diketiminate) moiety have been previously explored, and the methyl substitution in the NacNac backbone provided optimal results [54, 56]. For instance, the methyl group in the β-diketiminate led to effective destabilization of the HOMO, reducing the HOMO–LUMO gap and red-shifting the absorption profile [54–56]. Furthermore, the local energy decomposition (LED) based on the DLPNO-CCSD(T) method was performed for all systems at the singlet DFT-optimized geometry (using the most suitable functional, as discussed further) to provide insight into the metal-ligand interactions.

All ground-state geometry optimizations were performed using a DFT method and the def2-TZVP basis set [57–61]. The “def2” basis sets, developed by Ahlrichs and co-workers, were found to produce a good balance between computational efficiency and accuracy [59]. Initially, we used the X-ray geometry of the Cu(I) parent complex to select the most appropriate functional for describing it. The following density functionals were explored: the BP86-D3(BJ) [62, 63], M06-L [62], TPSS-D3(BJ) [64], B3LYP/G-D3(BJ) [63, 65–67], PBE0-D3(BJ) [68, 69], O3LYP-D3(BJ) [70], TPSSh-D3(BJ) [64, 71], CAM-B3LYP-D3(BJ) [72], ωB97X-D3(BJ) [73], and ωB97M-D3(BJ) [74]. The D3(BJ) indicates that the Becke–Johnson dispersion correction was used [75]. The absence of negative vibrational frequencies confirmed that a minimum molecular structure was obtained.

To compare DFT and X-ray structures of the parent complex, three metrics were used. Specifically, RMSE (root mean square error) measured the Cartesian coordinates of all atoms, while MUE (mean unsigned error) and MSE (mean signed error) assessed interatomic distances. To minimize errors due to the X-ray positions of H atoms, the error parameters were evaluated with H atoms excluded.

To investigate the interaction between the substituted 2,2′-biquinoline N$^\wedge$N^R^ ligand and the remaining part of the complex, the local energy decomposition (LED) approach [76, 77] at the DLPNO-CCSD(T) level [78–80], as implemented in ORCA, was employed. Within this framework, the interaction energy ($\Delta E_{\text {int}}$) is decomposed as follows:

1 $$\begin{aligned} \Delta E_{\text {int}}&= \Delta E_{\text {el-prep}}^{\text {ref.}}+E_{\text {elstat}}^{\text {ref.}}+E_{\text {exch}}^{\text {ref.}}+\Delta E_{\text {non-dispersion}}^{\text {C-CCSD}}\nonumber \\&\quad +E_{\text {dispersion}}^{\text {C-CCSD}}+\Delta E_{\text {int}}^{\text {C-(T)}} \end{aligned}$$

Here, $\Delta E_{\text {el-prep}}^{\text {ref.}}$ is the electronic preparation energy required to deform the fragments from their reference states to their states in the complex. $E_{\text {elstat}}^{\text {ref.}}$ is the electrostatic interaction; $E_{\text {exch}}^{\text {ref.}}$ is the exchange (Pauli) repulsion; $\Delta E_{\text {non-dispersion}}^{\text {C-CCSD}}$ covers nondispersive correlation, mainly polarization and charge-transfer effects; $E_{\text {dispersion}}^{\text {C-CCSD}}$ is the dispersion term; and $\Delta E_{\text {int}}^{\text {C-(T)}}$ is the perturbative triples contribution.

The def2-SVP basis set and the corresponding auxiliary basis set [57, 58] were used for all DLPNO-CCSD(T) calculations.

ii) TD-DFT protocol and the calculation of electronic excited-state properties: In this second main task, the UV–vis absorption spectra and the nature of electronic transitions for the parent complex were used as reference, since both types of data are experimentally available. Subsequently, the series of substituted Cu(I) complexes was also investigated.

Experimentally, the MLCT absorption maximum of the parent Cu(I) system was reported to be around 800 nm in toluene solution [54, 81]. Since the experimental UV–Vis absorption spectrum was obtained in solution, solvent effects were considered through the implicit Conductor-like Polarizable Continuum Model (C-PCM) [82, 83] in the calculations performed after the benchmark studies. This transition plays an essential role in the design of photosensitizers [84–86]; hence, it was used to select the most suitable density functional for photophysical properties. Five different functionals were assessed: O3LYP, B3LYP, CAM-B3LYP, LC-ωPBE [87–90], and ωB97X-D. After selecting the most suitable functional, TD-DFT calculations were carried out using the def2-TZVP basis set and the optimized ground-state geometries obtained in the presence of implicit toluene solvent. The 30 low-lying singlet electronic states were included.

To help characterize the nature of the electronic excited states, the TD-DFT results obtained at the TD-DFT/CAM-B3LYP/def2-TZVP/C-PCM(toluene) level of theory were analyzed using the TheoDORE program [91]. For this purpose, each complex was partitioned into three fragments corresponding to the copper(I) center (M), the N$^\wedge$N^R^ ligand (L), and the NacNac^Me^ ligand (L’). The excited-state character was quantified through fragment-based charge-transfer descriptors derived from charge-transfer numbers and fragment contributions, which describe the electronic redistribution between the predefined fragments upon excitation. Such fragment-based analyses have been successfully employed for a broad range of chemical systems to characterize the nature of excited states and to distinguish localized excitations from charge-transfer contributions [92–98]. These descriptors were used to quantify and assign metal-to-ligand charge transfer (MLCT), ligand-to-ligand charge transfer (LLCT), ligand-centered (LC), and metal-centered (MC) contributions to each excited state. Natural transition orbitals (NTOs) [99, 100] were additionally computed for selected relevant transitions, thereby complementing the fragment-based charge-transfer characterization obtained using TheoDORE. All DFT, TD-DFT, and NTO calculations were performed with the ORCA program (version 6.0.1) [101].

## Results and discussion

### Density functional for ground state

First of all, the best density functional was selected through the RMSE, MUE, and MSE metrics conducted using the Cu(N$^\wedge$N^H^)(NacNac^Me^) parent complex X-ray structure and the gas-phase DFT geometries. Figure 2 presents these results.

Analyzing the error metrics in Fig. 2, it is observed that the functional with the lowest MSE value is ωB97M-D3(BJ), with a value of −0.0496 Å. This indicates that this functional underestimates interatomic distances to a lesser extent. For the RMSE and MUE metrics, the lowest values are obtained with the M06-L functional: 0.6703 Å and 0.2462 Å, respectively. CAM-B3LYP-D3(BJ) follows, yielding RMSE and MUE values of 0.6816 Å and 0.2604 Å.

When comparing the CAM-B3LYP-D3(BJ) and M06-L functionals, the meta-GGA M06-L is, in principle, expected to offer lower computational demands due to the absence of exact exchange contributions. However, its extensive empirical parametrization is optimized to reproduce a broad set of molecular properties. This can result in less continuous and more irregular potential energy surfaces (PES), particularly in regions involving bond rearrangements or significant electronic reorganization [59, 102–104]. Such topographical irregularities of the PES often hinder self-consistent field (SCF) convergence and geometry optimizations. This behavior is especially pronounced in transition-metal systems where multiple near-degenerate electronic states are accessible [105]. These convergence instabilities may require additional corrective measures, including alternative SCF algorithms, level shifting, or tighter numerical integration grids. Ultimately, such needs diminish the nominal computational efficiency of the M06-L functional.

**Fig. 3:**
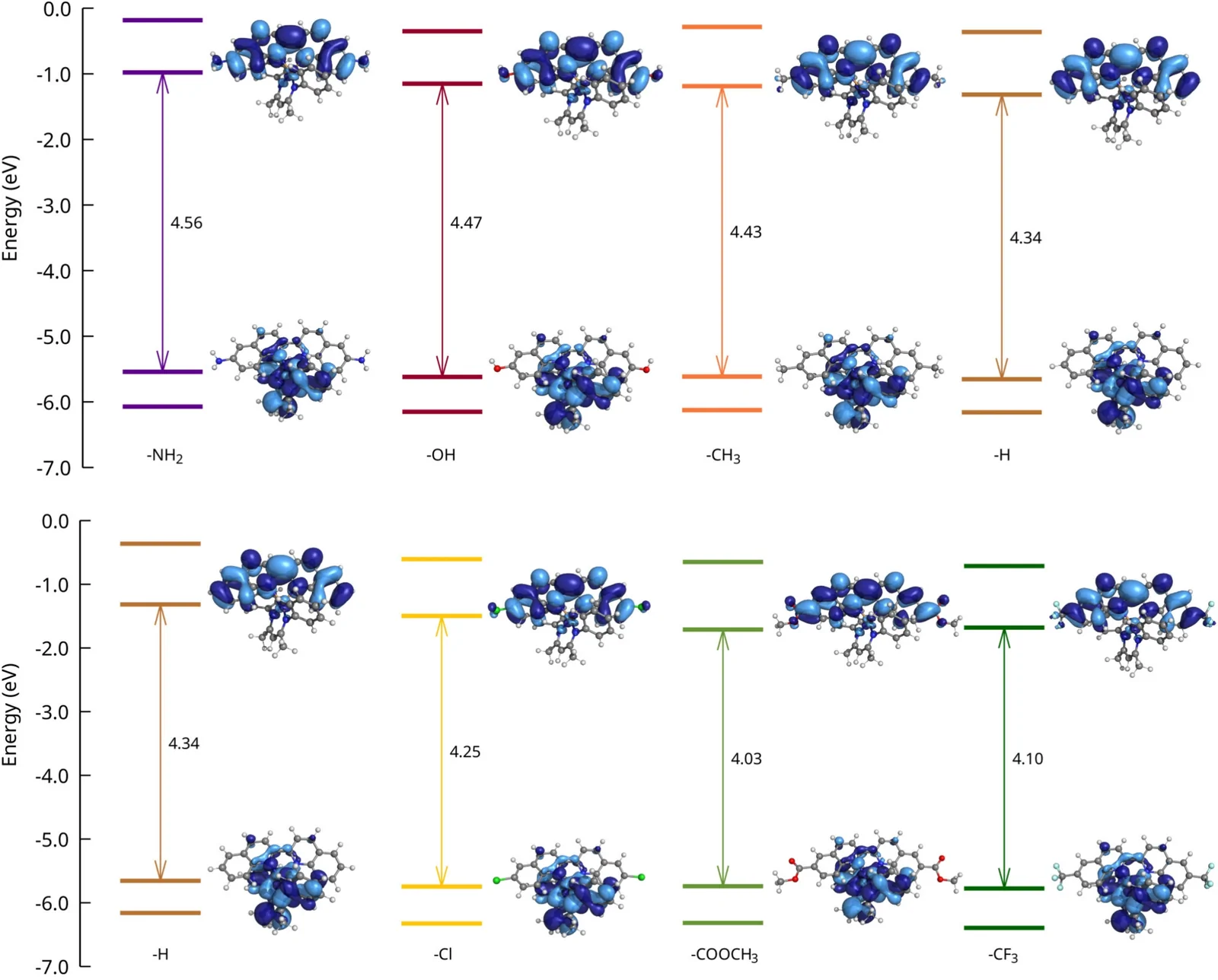
DFT/CAM-B3LYP-D3(BJ)/def2-TZVP/C-PCM(toluene) ground-state molecular orbital (MO) diagrams for the Cu(I) complexes featuring **a** electron-donating and **b** electron-withdrawing substituents. The substituent groups are indicated at the bottom of each diagram. For each complex, the energies of the HOMO-1, HOMO, LUMO, and LUMO+1 orbitals are presented, together with the HOMO–LUMO energy gap (ΔE, in eV). The corresponding isosurfaces of the frontier molecular orbitals, HOMO and LUMO, are also shown

In contrast, CAM-B3LYP-D3(BJ), a long-range-corrected functional, uses 65 %. long-range Hartree–Fock exchange. This approach better balances short- and long-range exchange, resulting in smoother, more consistent potential energy surfaces. As a result, geometry optimizations converge more reliably, and stationary points are easier to find. Here, the convergence instabilities with M06-L made CAM-B3LYP-D3(BJ) the more robust and practical choice for this study.

Given these advantages, we selected the DFT/def2-TZVP/CAM-B3LYP-D3(BJ) level of theory as the most appropriate approach for describing the ground-state properties. We therefore use this approach in our investigations on Cu(I) complexes.

### Substituent effects on ground-state properties

The DFT/CAM-B3LYP/def2-TZVP protocol was first validated against the X-ray crystal structure of the parent complex. After selecting the appropriate computational protocol, the molecular geometries of all complexes were optimized using the DFT/CAM-B3LYP/def2-TZVP/C-PCM(toluene) level of theory, which was subsequently used to compute their molecular orbital (MO) diagrams (Fig. 3).

Analysis of the frontier orbital energies (Table S1, Supplementary Information) reveals a clear substituent-dependent trend. As the electron-withdrawing character of the R-group increases, the HOMO–LUMO energy gap systematically decreases. This effect arises predominantly from the stabilization of the LUMO, while the HOMO remains essentially unchanged across the series. The resulting narrowing of the energy gap suggests that the corresponding electronic transitions are expected to shift toward lower energies, i.e., further into the red region of the spectrum. This trend, however, requires confirmation through excited-state calculations, as discussed in Sect. TD-DFT functional selection: the parent complex UV–Vis.

**Fig. 4:**
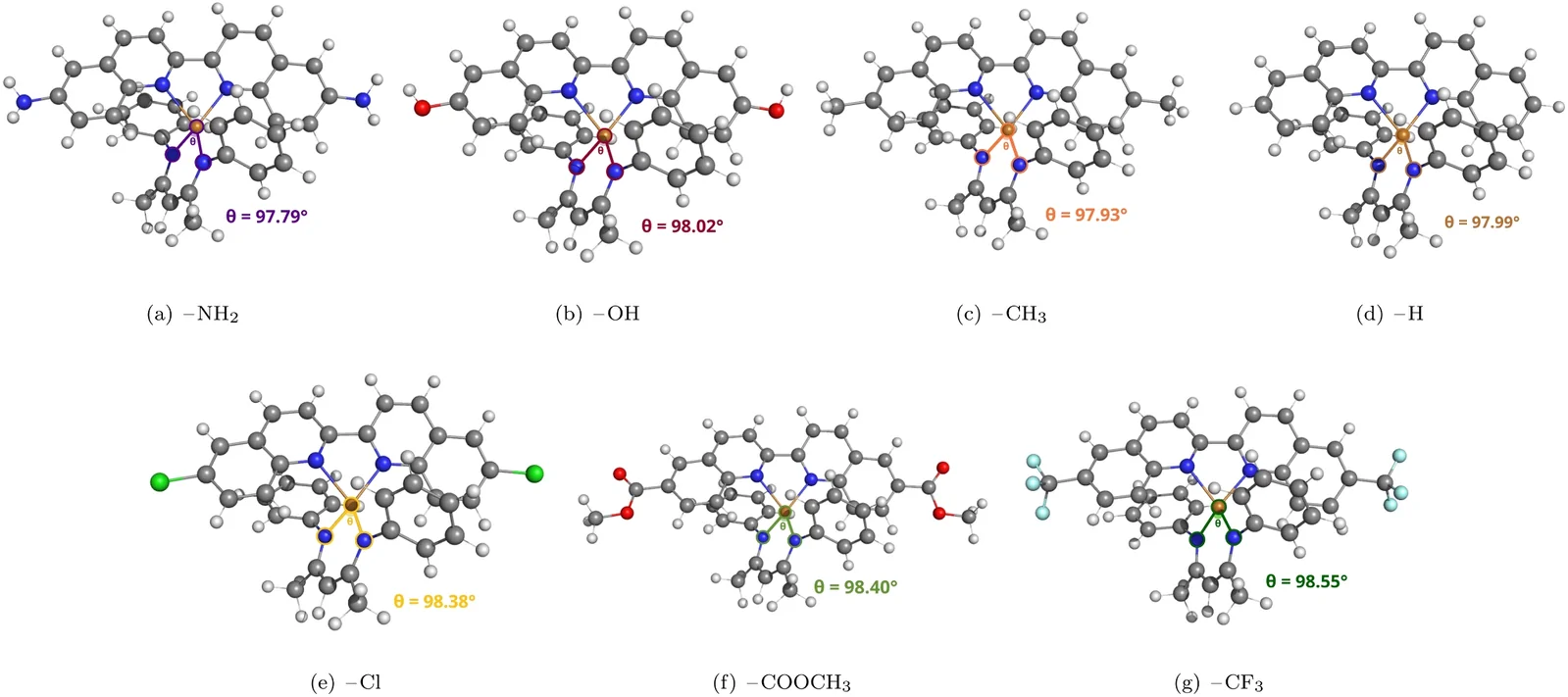
Optimized ground-state geometries of the seven Cu(N$^\wedge$R)(NacNac^Me^) complexes, highlighting the NacNac bite angle for each substituent (R = **a** -NH$_2$, **b** -OH, **c** -CH$_3$, **d** -H, **e** -Cl, **f** -COOCH$_3$, and **g** -CF$_3$), at DFT/CAM-B3LYP-D3(BJ)/def2-TZVP/C-PCM(toluene) level of theory. The bite angle is indicated directly on each 3D optimized structure

**Table 1:** Components of the LED analysis of the interaction energy of the fragments, values in kcal mol$^{-1}$ (def2-SVP/TightPNO level of theory)

	$\Delta E^{ref.}_{el-prep}$	$\Delta E^{ref.}_{elstat}$	$\Delta E^{ref.}_{exch}$	$\Delta E^{C-CCSD}_{non-disp.}$	$\Delta E^{C-CCSD}_{disp.}$	$\Delta E^{C-(T)}_{int}$	Sum
R = NH$_2$	448.13	−405.43	−59.99	−17.05	−21.31	−5.70	−61.36
R = OH	448.83	−404.36	−60.45	−17.04	−21.29	−5.74	−60.05
R = CH$_3$	447.10	−403.41	−59.99	−17.00	−21.23	−5.68	−60.21
R = H	447.73	−403.42	−60.22	−17.02	−21.11	−5.72	−59.76
R = Cl	451.61	−404.86	−61.44	−17.03	−22.06	−5.91	−59.68
R = COOCH$_3$	450.39	−403.68	−61.24	−17.10	−22.13	−5.89	−59.66
R = CF$_3$	456.45	−407.21	−62.67	−17.58	−22.30	−6.10	−59.42

This behavior is consistent with previous studies, which demonstrated that modifications on the N$^\wedge$N ligand primarily influence the LUMO energy [56]. Because the Cu(I) coordination environment and the NacNac ligand are identical in all complexes, the donor fragment is essentially preserved, and only minor variations in the HOMO energy are observed. This indicates that substituent effects are concentrated mainly on the acceptor N$^\wedge$N ligand rather than on the metal-centered donor fragment.

Beyond the orbital energies, the spatial distribution of the frontier orbitals provides insight into the nature of the low-energy electronic transitions. The HOMO is localized primarily on the Cu center and the NacNac ligand, whereas the LUMO is mainly located on the biquinoline fragment. This spatial separation between the donor (metal/NacNac) and acceptor (biquinoline) moieties is consistent with the mixed metal-to-ligand and ligand-to-ligand charge-transfer character (MLCT/LLCT) commonly reported for heteroleptic Cu(I) diimine complexes [106, 107] and is further supported by the excited-state analyses presented in Sect. TD-DFT functional selection: the parent complex UV–Vis. Within this framework, the Cu(I)/NacNac fragment acts as the electron donor, while substituent modifications on the N$^\wedge$N ligand mainly tune the acceptor orbital energies, particularly the LUMO, thereby controlling the HOMO–LUMO gap and the energies of the low-energy electronic transitions across the series.

In addition to the molecular-orbital analysis, the bite angles of the NacNac^Me^ ligand were examined across the full series of substituted complexes, as shown in Fig. 4. A general increase in the bite angle is observed toward the electron-withdrawing end of the series, resulting in ground-state coordination geometries that are slightly closer to the ideal tetrahedral arrangement. This trend demonstrates that the electronic nature of the substituent affects the ground-state coordination geometry around the Cu(I) center.

While the overall trend is uniform, one notable deviation is observed for the hydroxyl-substituted complex. Its bite angle is larger than those of the -CH$_3$ and -H derivatives, despite -OH being more electron-donor. This deviation suggests that additional factors may influence the bite angle in this specific case.

Overall, the bite-angle analysis indicates that electron-withdrawing substituents lead to a slightly wider NacNac^Me^ bite angle and a ground-state coordination environment closer to tetrahedral geometry. To relate this structural trend to the ground-state metal–ligand interaction strength, a local energy decomposition (LED) analysis at the DLPNO-CCSD(T) level was performed.

As shown in Table 1, the total interaction energy becomes more negative as the substituent becomes more electron-donating, following the trend: CF$_3$ < COOCH$_3$ < Cl < H < OH < CH$_{3}$ < NH$_{2}$. The interaction energy ranges from −59.42 kcal mol$^{-1}$ for the strongly electron-withdrawing CF$_3$ group to −61.36 kcal mol$^{-1}$ for the electron-donating NH$_2$ substituent, indicating stronger overall metal–ligand interactions for donor groups. As also observed for the bite angles, the hydroxyl substituent slightly deviates from the expected donor–withdrawer ordering, indicating that substituent effects cannot be described solely by a simple inductive model.[108] In addition to inductive effects, resonance (mesomeric) contributions, π-conjugation, and through-space electrostatic interactions, associated with local field effects generated by substituent dipoles, must also be considered.[109, 110] In particular, substituents such as OH can donate electron density via π-delocalization of lone pairs into the ligand framework, enhancing the covalent character of the metal–ligand interaction beyond what is expected from inductive effects alone.[111, 112] Overall, these results are consistent with the observed trend, in which electron-donating groups increase the covalent character of the Cu–ligand interaction, leading to stronger binding in the ground state.

Inspection of the individual LED components provides a more nuanced picture. The exchange contribution ($\Delta E^\mathrm {ref.}_\textrm{exch}$) becomes increasingly stabilizing toward the electron-withdra-wing end of the series, as seen for the CF$_3$ substituent. This trend indicates increased metal–ligand orbital overlap, a feature commonly associated with enhanced covalency and often linked to the nephelauxetic effect[113, 114]. However, the total interaction energy follows the opposite ordering, being more stabilizing for electron-donating substituents. Such a trend indicates that the exchange stabilization is counterbalanced by other contributions, particularly preparation and electrostatic terms.

Thus, although the exchange term suggests a nephelauxe-tic-like increase in covalency for electron-withdrawing substituents, the overall LED balance cautions against a direct one-to-one interpretation. In general, the substituent dependence of the total interaction energy is governed primarily by the exchange term, with smaller contributions from dispersion and correlation. Electron-donating substituents increase electron density available for metal–ligand overlap, leading to more stabilizing interactions, while the electrostatic component remains nearly constant across the series, consistent with the unchanged formal charge distribution.

The hydroxyl substituent again appears as an outlier within the donor group. Its interaction energy (−60.05 kcal mol$^{-1}$) is slightly less stabilizing than expected relative to CH$_3$, suggesting that secondary effects such as local polarization or partial resonance withdrawal into the diimine system may influence the interaction. Overall, the LED results support the structural and molecular-orbital trends discussed above, with the electron-donating substituents strengthening the Cu–ligand interaction.

### TD-DFT functional selection: the parent complex UV–Vis

**Fig. 5:**
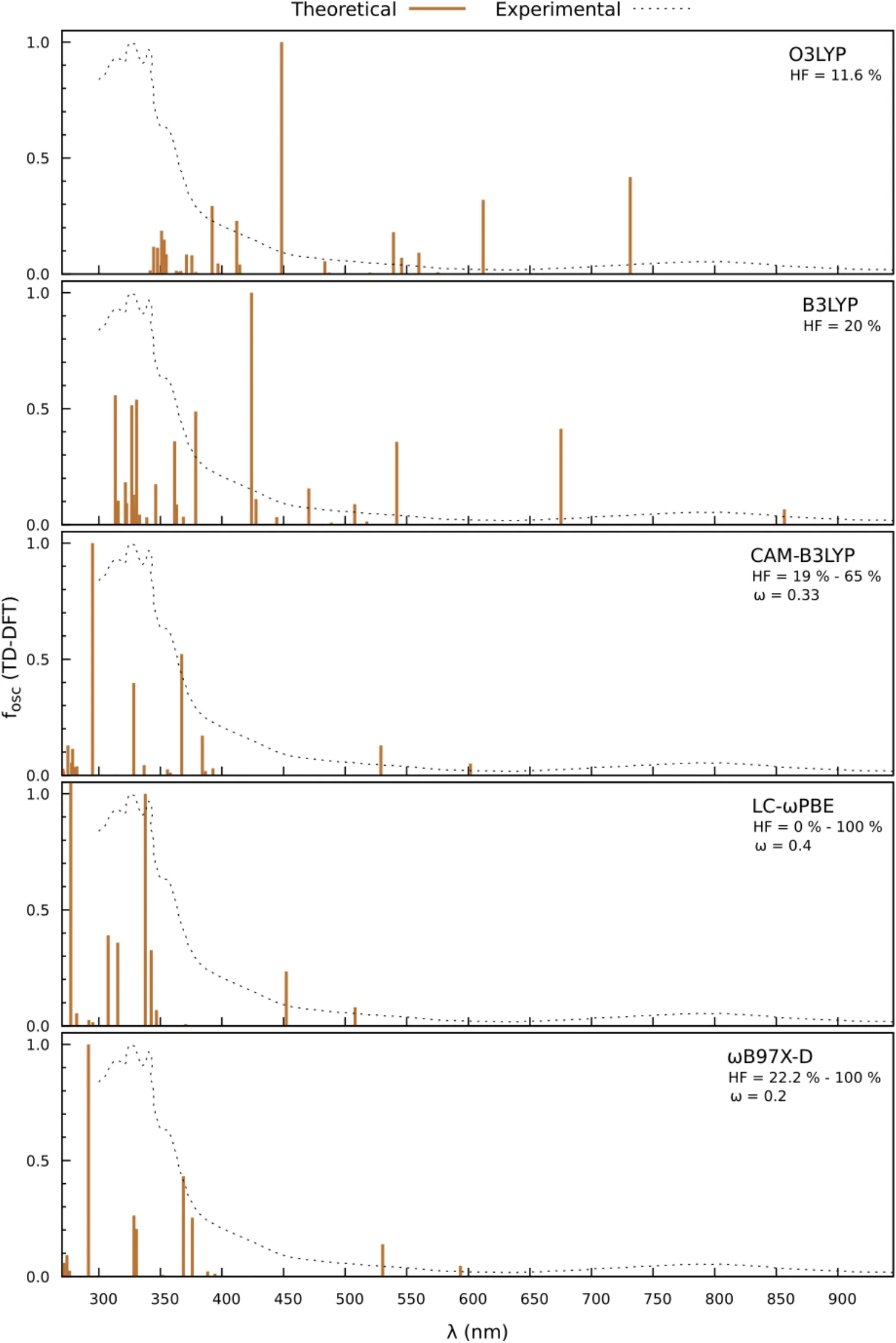
Parent Cu(N$^\wedge$N^H^)(NacNac^Me^) theoretical UV–Vis absorption spectra. Percentages of exact HF exchange are shown and, for range-separated hybrid functional, the ω range-separated parameter is also identified. In all calculations the def2-TZVP basis set and the C-PCM(toluene) model were applied

We next turn our attention to the excited-state properties of the complexes. To select an appropriate density functional for TD-DFT calculations, we explore a set of five functionals against the experimental UV–Vis absorption spectrum of the parent complex as reference data [54]. Figure 5 brings the results obtained using five density functionals. In all calculations, the def2-TZVP basis set and the C-PCM(toluene) model were applied.

Among the functionals tested, CAM-B3LYP and ωB97X-D most faithfully reproduced the overall spectral profile in both the MLCT and intraligand regions. The lowest-energy transitions computed with these two functionals occur at ca. 601.7 nm (CAM-B3LYP) and 593.6 nm (ωB97X-D), whereas the experimental MLCT band for the parent complex is observed near 796 nm [54]. Thus, both functionals produce a blue shift relative to experiment, but CAM-B3LYP (longer wavelength) provides a closer match. This result is consistent with the design of CAM-B3LYP: it combines moderate short-range HF exchange (19%) with a higher fraction (65%) at long range [72], and was shown by Yanai et al. to perform well for charge-transfer excitations that B3LYP severely underestimates [72]. For these reasons (better placement of the low-energy MLCT band and its well-behaved long-range exchange), CAM-B3LYP was chosen for subsequent TD-DFT analysis.

### Excited-state analyses of the parent complex

**Fig. 6:**
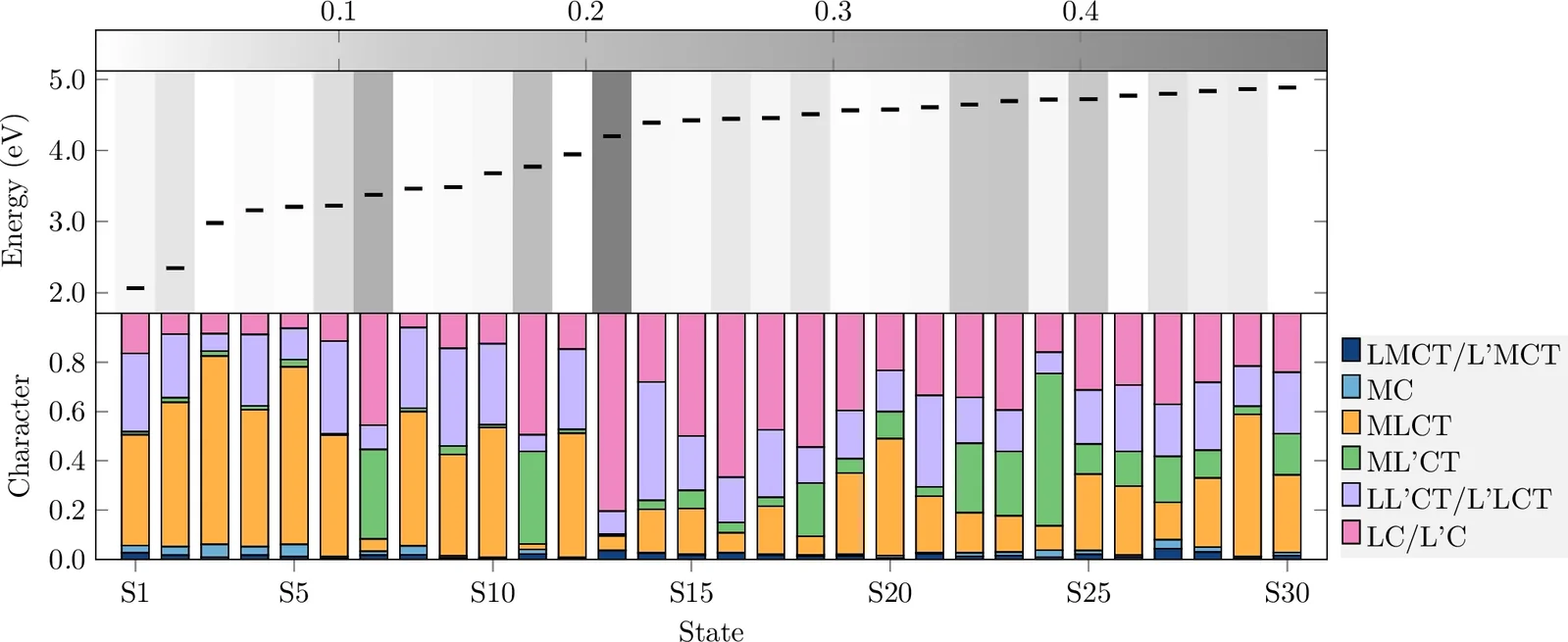
Energies and charge-transfer number analysis of the 30 lowest-energy electronic transitions calculated at the TD-DFT/CAM-B3LYP/def2-TZVP/C-PCM(toluene) level of theory, obtained from Löwdin population analysis, with the complex fragmented into the copper center (M), the N$^\wedge$N^H^ ligand (L), and the NacNac^Me^ ligand (L′). The intensity of the grayscale shading in the upper panel is proportional to the oscillator strength (*f*) of each transition

**Fig. 7:**
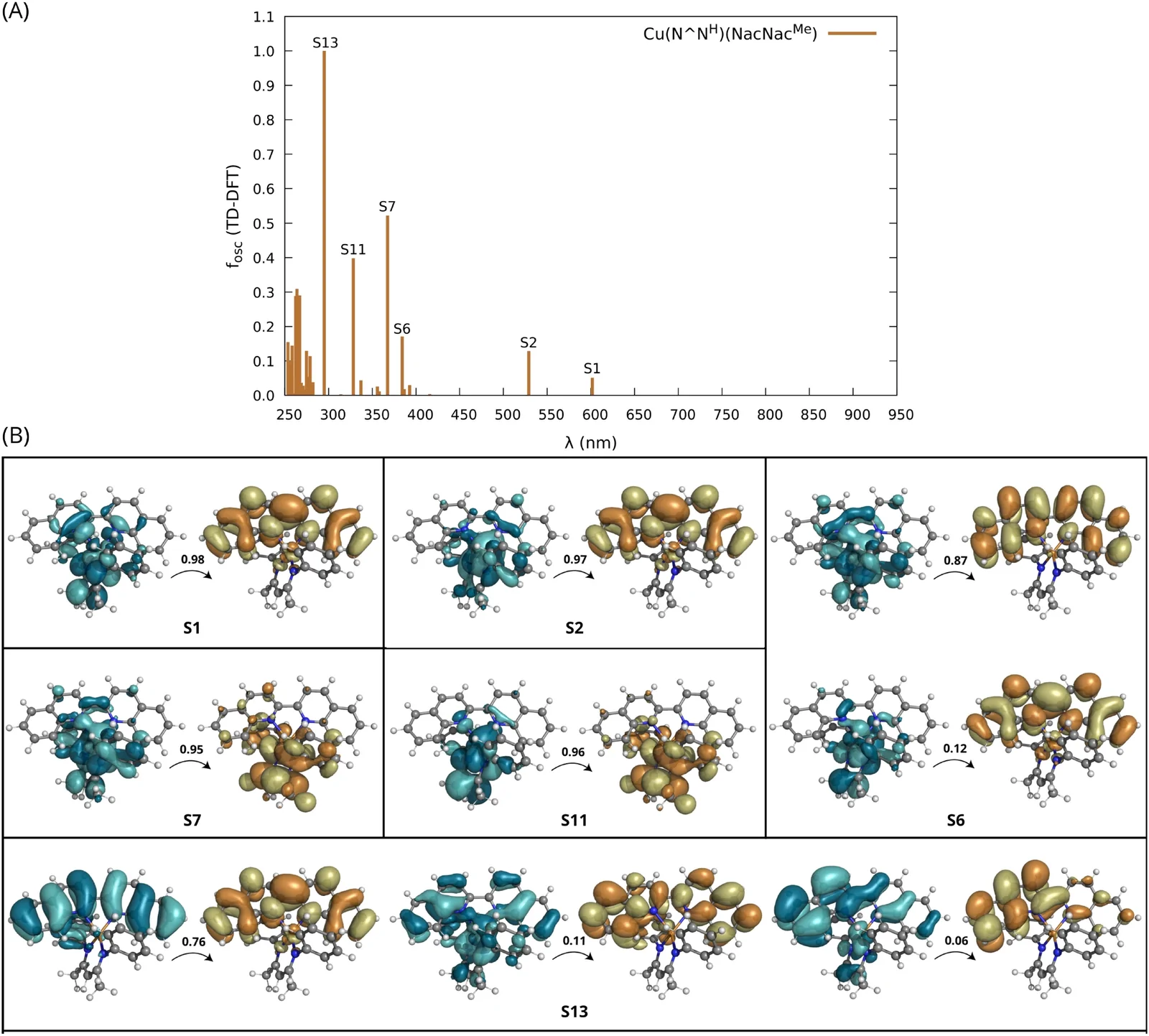
Cu(N$^\wedge$N^H^)(NacNac^Me^) data: **A** electronic absorption spectrum calculated at the TD-DFT/CAM-B3LYP/def2-TZVP/C-PCM(toluene) level of theory and **B** NTO pairs for selected transitions, with the weights indicated above the arrows. The labels below each set of NTOs indicate the corresponding excited states

To characterize the nature of the electronic excited states in greater detail, the 30 lowest singlet states of the parent complex were analyzed using the TheoDORE program (Fig. 6). The complex was partitioned into three fragments corresponding to the copper center (M), the $\mathrm {N^\wedge N^H}$ ligand (L), and the $\mathrm {NacNac^{Me}}$ ligand (L′). The fragment-based charge-transfer contributions were evaluated to characterize the redistribution of electron density between the predefined fragments upon excitation. Natural transition orbitals (NTOs) were analyzed for selected transitions to provide a complementary orbital-space representation of the excited-state character (Fig. 7).

The fragment-based excited-state character analysis shown in Fig. 6 quantitatively confirms that the lowest-energy excitations of the parent complex have mixed MLCT/LLCT character. For $S_1$ at 2.06  eV, the MLCT and interligand charge-transfer contributions are approximately 45 and 32%, respectively, whereas for $S_2$ at 2.34 eV, they amount to approximately 59 and 26%. More generally, the $S_1$–$S_6$ manifold contains 45–76% MLCT character, accompanied by 7–38% LL′CT/L′LCT contributions (see Table S5, Supplementary Information). Thus, the low-energy absorption cannot be described as a purely metal-to-ligand transition; rather, it involves electronic redistribution from the Cu/NacNac^Me^ donor region toward the N$^\wedge$N^H^ acceptor ligand, in agreement with the frontier-orbital picture.

At higher energies, the excited-state character becomes more heterogeneous. The transitions with the largest oscillator strengths are $S_{13}$ (f≈0.50), $S_7$ (f≈0.31), and $S_{11}$ (f≈0.26). The $S_7$ and $S_{11}$ states are mainly mixtures of ligand-centered excitation (approximately 45–49%) and ML′CT (approximately 36–38%), whereas $S_{13}$ is predominantly ligand-centered (approximately 81%). Metal-centered and ligand-to-metal contributions remain individually below approximately 6% throughout the 30-state manifold, indicating that these excitation classes play only a minor role in the calculated absorption spectrum. The analysis therefore distinguishes the low-energy mixed MLCT/LLCT band from the more intense, predominantly ligand-centered transitions at higher energies.

The NTOs in Fig. 7 provide a complementary orbital-space representation of the same electronic redistribution. The low-energy states display hole density distributed over the Cu/NacNac^Me^ donor region and electron density mainly on the N$^\wedge$N^H^ ligand, whereas the more intense higher-energy states show increasingly ligand-localized character.

Together, the NTO analysis and the excited-state character descriptors obtained with the TheoDORE program provide mutually consistent evidence for the mixed MLCT/LLCT assignment of the low-energy absorption. These results further support the use of CAM-B3LYP for the description of the charge-transfer excitations in these Cu(I) chromophores, in line with its known efficacy for long-range charge-transfer states [72, 115]. At the same time, the persistent ∼0.5 eV blue shift relative to experiment suggests that residual errors, such as limitations of the solvent model, vibronic coupling, or remaining functional inaccuracies, may still influence the absolute transition energies. Accordingly, CAM-B3LYP/def2-TZVP/C-PCM(toluene) was adopted for the subsequent analysis of substituent effects across the Cu(N$^\wedge$N^R^)(NacNac^Me^) series.

### Substituent effects on excited-state properties

Having established the nature of the low-energy excited states of the parent complex, we next examine how systematic substitution on the N$^\wedge$N^R^ ligand modifies their electronic and spectroscopic properties. To establish a direct structure–property relationship, the ground-state electronic and structural descriptors discussed in Sect. Substituent effects on ground-state properties were compared with the vertical $S_0\rightarrow S_1$ excitation properties calculated at the $S_0$ equilibrium geometries.

In the $S_0$ state, substituent effects are concentrated primarily on the LUMO, which is localized on the N$^\wedge$N^R^ ligand, whereas the HOMO, associated mainly with the Cu/NacNac^Me^ fragment, remains comparatively unchanged across the series. Consequently, the HOMO–LUMO gap decreases from 4.56  eV for $R={\text {NH}_2}$ to 4.03  eV and 4.10  eV for $R={\text {COOCH}_3}$ and $R={\text {CF}_3}$, respectively. This ground-state electronic trend is directly reflected in the vertical $S_0\rightarrow S_1$ excitation energies. Electron-donating substituents produce higher-energy $S_1$ states, whereas electron-withdrawing substituents stabilize the acceptor orbital and shift the low-energy absorption band toward longer wavelengths.

**Fig. 8:**
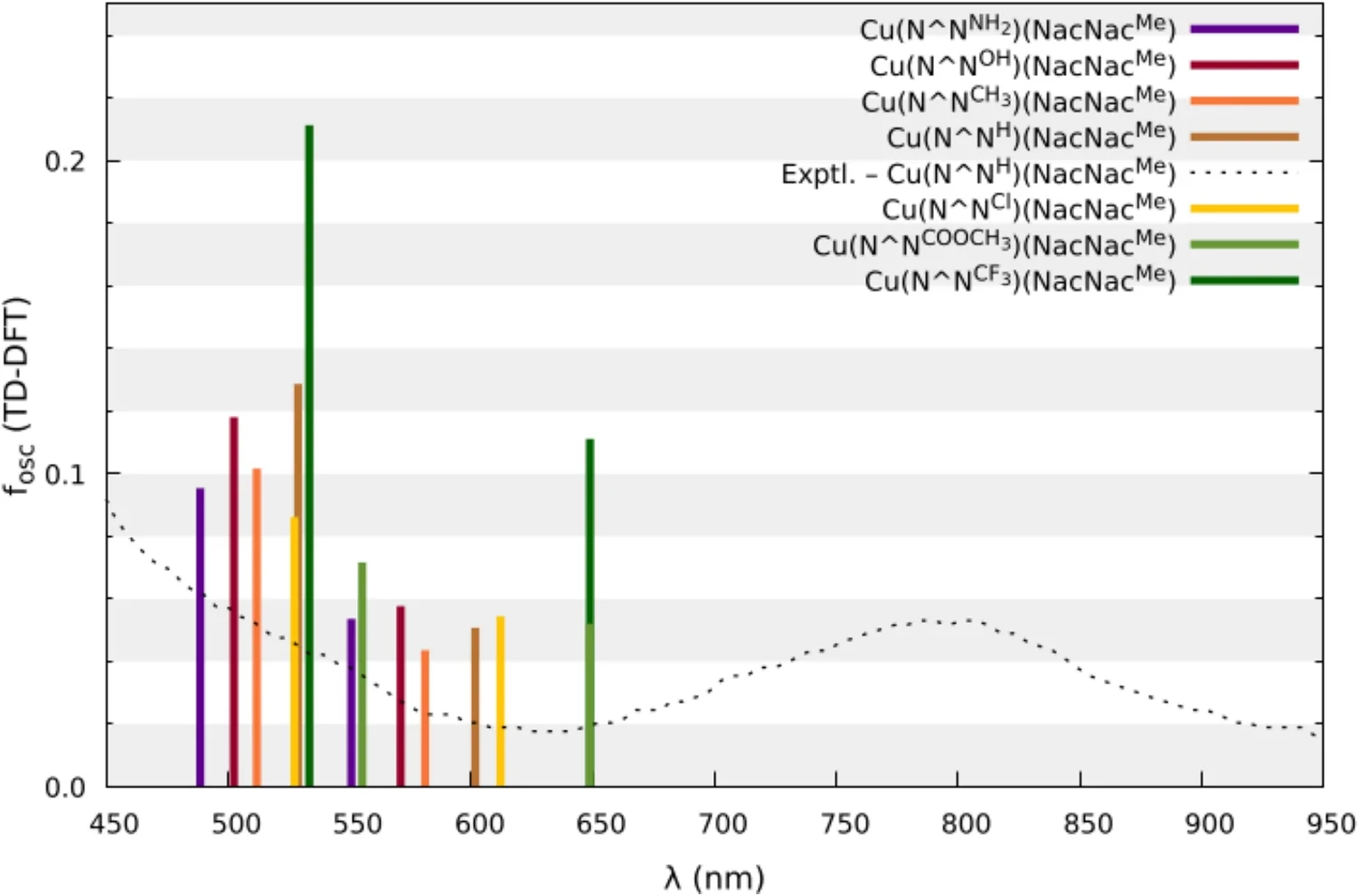
TD-DFT/CAM-B3LYP/def2-TZVP/C-PCM(toluene) UV-Vis absorption spectra in the MLCT region (450–950 nm) for all complexes: Cu(N$^\wedge \text {N}^{\text {NH}_2}$)(NacNac^Me^), Cu(N$^\wedge$N^OH^)(NacNac^Me^), Cu(N$^\wedge \text {N}^{{\text {CH}}_3}$)(NacNac^Me^), Cu(N$^\wedge$N^H^)(NacNac^Me^), Cu(N$^\wedge$N^Cl^)(NacNac^Me^), Cu(N$^\wedge \text {N}^{{\text {COOCH}}_3}$)(NacNac^Me^), and Cu(N$^\wedge \text {N}^{{\text {CF}}_3}$)(NacNac^Me^). The experimental spectrum of Cu(N$^\wedge$N^H^)(NacNac^Me^) in the MLCT region is also included for comparison

Based on the importance of the low-energy charge-transfer band for these Cu(I) systems, the calculated spectra of all complexes were compared in the 450–950 nm region at the TD-DFT/CAM-B3LYP/def2-TZVP/C-PCM(toluene) level of theory (Fig. 8). The most pronounced red shifts are obtained for the strongly electron-withdrawing COOCH$_3$ and CF$_3$ substituents, for which the $S_0\rightarrow S_1$ transition occurs near 650 nm. In contrast, the electron-donating substituents NH$_2$, OH, and CH$_3$ shift this transition to higher energies. Thus, among the ground-state descriptors considered, LUMO stabilization and the resulting reduction of the HOMO–LUMO gap provide the most direct explanation for the substituent-dependent $S_1$ energies.

Figure 8 focuses on the 450–950 nm region, in which only the $S_0$ → $S_1$ and $S_0$ → $S_2$ transitions are observed for all complexes. These transitions constitute the low-energy band assigned experimentally to metal-to-ligand charge-transfer excitations. Although substituent variation provides an effective strategy for tuning the electronic properties of the N$^\wedge$N ligand, the photophysical behavior of these Cu(I) complexes arises from the combined effects of the heteroleptic Cu(I)/NacNac/N$^\wedge$N coordination framework and the electronic modulation introduced by the substituents. The coordination environment defines the donor–acceptor electronic structure underlying the charge-transfer character of the low-energy excited states, whereas substituent effects primarily tune their relative energies and spectral positions. To provide a more comprehensive summary of the calculated UV–Vis properties, Table 2 reports the energies, wavelengths, oscillator strengths, and excited-state character contributions for the $S_0$ → $S_1$ and $S_0$ → $S_2$ electronic transitions. In addition to the fragment-based excited-state character discussed above, the quantitative TheoDORE descriptors $q_{\textrm{CT}}$ and $d_{eh}$ provide further insight into the extent and spatial distribution of charge transfer across the substituted series. The complete calculated spectra and the calculated UV–Vis properties are provided in the Supplementary Information, Figs. S1–S7 and Tables S2-S8.

**Table 2:** Calculated UV–Vis absorption data and excited-state descriptors obtained from charge-transfer number analysis performed with the TheoDORE program for the two lowest-energy singlet transitions ($S_1$ and $S_2$) associated with the visible charge-transfer band of the Cu(N$^\wedge$N^R^)(NacNac^Me^) complexes. The vertical excitation energies ($E_{\mathrm {exc.}}$), corresponding absorption wavelengths (λ), and oscillator strengths ($f_{\mathrm {osc.}}$) were calculated at the TD-DFT/CAM-B3LYP/def2-TZVP/C-PCM(toluene) level of theory. The charge-transfer number analysis was performed based on Löwdin population analysis, with each complex partitioned into the copper center (M), the N$^\wedge$N^R^ ligand (L), and the NacNac^Me^ ligand (L′). $q_{\textrm{CT}}$ denotes the TheoDORE charge-transfer number, while $d_{eh}$ represents the root-mean-square electron–hole separation distance. “Others” represents the combined LMCT/L′MCT, MC, and ML′CT contributions. For comparison, the experimental MLCT absorption band of the parent complex ($R={H}$) was reported at approximately 796 nm (1.56 eV) [54]

	State	λ (nm)	$E_{\mathrm {exc.}}$ (eV)	$f_{\mathrm {osc.}}$	$q_{\textrm{CT}}$	$d_{eh}$ (Å)	TheoDORE character contributions			
							MLCT	LL′CT/L′LCT	LC/L′C	Others
R= NH$_2$	S1	550.7	2.25	0.027	0.806	4.221	46.0%	30.9%	16.5%	6.6%
	S2	488.7	2.54	0.048	0.876	4.076	60.8%	23.3%	8.8%	7.1%
R= OH	S1	571.1	2.17	0.027	0.809	4.222	45.0%	32.1%	16.1%	6.8%
	S2	502.5	2.47	0.056	0.878	4.078	59.8%	24.5%	8.5%	7.2%
R= CH$_3$	S1	581.0	2.13	0.026	0.805	4.225	45.7%	31.1%	16.5%	6.7%
	S2	512.0	2.42	0.062	0.881	4.122	59.3%	25.2%	8.4%	7.1%
R= H	S1	601.7	2.06	0.025	0.807	4.220	45.1%	31.9%	16.3%	6.7%
	S2	529.0	2.34	0.064	0.881	4.118	58.6%	26.0%	8.5%	6.9%
R= Cl	S1	612.0	2.03	0.034	0.818	4.363	41.4%	36.4%	15.4%	6.8%
	S2	527.5	2.35	0.055	0.875	4.113	59.6%	24.3%	8.7%	7.4%
R= COOCH$_3$	S1	648.7	1.91	0.037	0.823	4.606	39.9%	38.6%	15.1%	6.4%
	S2	555.2	2.23	0.050	0.876	4.319	59.4%	24.7%	8.9%	7.0%
R= CF$_3$	S1	648.8	1.91	0.036	0.813	4.426	40.3%	36.7%	15.8%	7.2%
	S2	533.4	2.32	0.069	0.876	4.198	57.4%	26.4%	8.6%	7.6%

The quantitative data in Table 2 reveal a clear relationship between substituent electronics and the vertical $S_1$ properties. For the electron-donating substituents R= NH$_2$, OH, and CH$_3$, the $S_1$ excitation energies range from 2.25 eV to 2.13 eV, corresponding to wavelengths between 550.7 nm and 581.0 nm. The parent complex displays an intermediate value of 2.06 eV (601.7 nm), while the electron-withdrawing Cl, COOCH$_3$, and CF$_3$ substituents lower the $S_1$ energy to 2.03 eV for Cl and 1.91 eV for both COOCH$_3$ and CF$_3$. Overall, substitution produces a red shift of approximately 98 nm between the NH$_2$- and CF$_3$-substituted complexes.

In contrast to the pronounced variation in excitation energy, the $S_1$ oscillator strengths remain within the relatively narrow range of 0.025–0.037 and do not exhibit a strictly monotonic dependence on the electron-donating or electron-withdrawing character of the substituent. Thus, substitution on the N$^\wedge$N^R^ ligand predominantly controls the spectral position of the low-energy transition rather than producing a substantial systematic enhancement of its intensity.

The fragment-based excited-state character analysis performed with the TheoDORE program further demonstrates that substitution modifies not only the energy but also the composition of the $S_1$ state. For the electron-donating derivatives, the MLCT contribution is approximately 45–46%, while the LL′CT/L′LCT contribution is approximately 31–32%. Toward the electron-withdrawing end of the series, the MLCT contribution decreases to approximately 40% for the COOCH$_3$ and CF$_3$ derivatives, whereas the interligand charge-transfer contribution increases to approximately 37–39%. The ligand-centered contribution remains comparatively constant at approximately 15–17 %. Therefore, the red shift induced by electron-withdrawing substituents is accompanied by a redistribution of the $S_1$ character toward a more pronounced interligand charge-transfer contribution, although the state retains mixed MLCT/LLCT character throughout the series. This redistribution is further reflected in the quantitative charge-transfer descriptors discussed below.

The quantitative TheoDORE descriptors provide further insight into the nature of the substituent effects on $S_1$. The charge-transfer number, $q_{\textrm{CT}}$, remains relatively similar across the series, ranging from 0.805–0.809 for the electron-donating substituents and the parent complex to 0.818, 0.823, and 0.813 for the Cl, COOCH$_3$, and CF$_3$ derivatives, respectively. Thus, substitution produces only a modest increase in the overall charge-transfer extent for the electron-withdrawing derivatives, with COOCH$_3$ showing the largest value. In contrast, the root-mean-square electron–hole separation, $d_{eh}$, shows a more pronounced variation. Whereas the electron-donating derivatives and the parent complex exhibit values of approximately 4.22 Å, $d_{eh}$ increases to 4.36 Å  for Cl, 4.61 Å  for COOCH$_3$, and 4.43 Å  for CF$_3$. These results indicate that electron-withdrawing substitution is associated with a modest increase in the spatial separation of the electron and hole, particularly for COOCH$_3$, while the overall charge-transfer magnitude remains comparatively stable. This behavior is consistent with the observed redistribution of $S_1$ character from MLCT toward LL′CT/L′LCT across the series.

The $S_2$ states are less sensitive to substitution in terms of their electronic character. They retain predominantly MLCT character (57–61%), accompanied by 23–26% LL′CT/L′LCT character, across all complexes. Their oscillator strengths, ranging from 0.048 to 0.069, are consistently larger than those of $S_1$. Consistently, the $q_{\textrm{CT}}$ values remain within a narrow range of 0.875–0.881, while $d_{eh}$ varies from approximately 4.08 to 4.32 Å. These results show that the substituent effect is most clearly expressed in the energy and MLCT/LLCT balance of the lowest excited state, whereas the character of $S_2$ remains more uniform across the series.

NTO analysis confirms the expected transition character across the series (Figs. S1–S7, Supplementary Information). The dominant low-energy excitations are of mixed metal-to-ligand and ligand-to-ligand charge-transfer character (MLCT/LLCT).

Comparison with the ground-state LED results shows that the total metal–ligand interaction energy does not follow the same ordering as the $S_1$ excitation energy. The total interaction is generally more stabilizing for electron-donating substituents, whereas the largest $S_1$ red shifts are observed for electron-withdrawing groups. Therefore, the strength of the total ground-state metal–ligand interaction is not the primary descriptor controlling the low-energy absorption. Instead, the $S_1$ energy is most directly associated with substituent-induced LUMO stabilization and narrowing of the HOMO–LUMO gap. The more stabilizing exchange contribution observed for electron-withdrawing substituents provides complementary information on changes in metal–ligand overlap, but it should be regarded as a secondary electronic-structure effect rather than the direct origin of the spectral shift.

Within the scope of the present calculations, substitution on the N$^\wedge$N^R^ ligand is shown to be an effective strategy for tuning the LUMO energy and, consequently, the calculated wavelength of the low-energy MLCT/LLCT absorption band. The bite-angle and LED analyses additionally characterize substituent-dependent variations in the ground-state coordination geometry and metal–ligand interactions. However, these ground-state descriptors do not directly establish excited-state stability or the propensity for geometric flattening.

Taken together, the ground- and excited-state calculations provide a coherent framework for understanding how substituent electronics modulate the ground-state geometry, frontier-orbital energies, metal–ligand interactions, and calculated absorption properties of these Cu(I) complexes. The TD-DFT calculations and the excited-state characterization performed using the TheoDORE program demonstrate that these modifications directly affect the position and composition of the low-energy MLCT/LLCT absorption band. These results therefore provide structure–property relationships for tuning the optical response of heteroleptic Cu(I) chromophores.

## Conclusion

In this work, we carried out a systematic computational investigation of a series of heteroleptic Cu(I) complexes of the type Cu(N$^\wedge$N^R^)(NacNac^Me^), focusing on the influence of R substituents on the N$^\wedge$N ligand. The combination of DFT, TD-DFT, and fragment-based charge-transfer analysis performed using the TheoDORE program, including quantitative charge-transfer descriptors, and DLPNO-CCSD(T)/LED analyses provided a comprehensive understanding of how substituent electronics influence molecular structure, frontier orbital energetics, metal–ligand interactions, and MLCT electronic transitions across this family of Cu(I) complexes.

The benchmarking study indicated that the CAM-B3LYP functional offers the best compromise between structural accuracy, convergence behavior, and description of charge-transfer excitations, and was therefore selected as the reference level of theory for the full series.

Frontier orbital analysis revealed that substituent effects act primarily on the LUMO, which is localized on the N$^\wedge$N ligand, while the HOMO remains largely unaffected due to its metal/NacNac character. As a result, electron-withdrawing substituents progressively reduce the HOMO–LUMO gap, leading to systematic red shifts of the MLCT transitions. This trend was confirmed by the TD-DFT absorption spectra, where the strongest electron-withdrawing groups (-CF$_3$ and -COOCH$_3$) produced the lowest-energy transitions in the series.

The excited-state analysis further showed that the lowest-energy absorption involves mixed MLCT/LLCT character across the series. Quantitative analysis obtained with TheoODRE indicated that the charge-transfer number, $q_{\textrm{CT}}$, remains relatively similar among the complexes, whereas the root-mean-square electron–hole separation, $d_{eh}$, exhibits a more pronounced increase for the electron-withdrawing derivatives, particularly for -COOCH$_3$. These results indicate that substitution modifies the spatial distribution and balance of charge transfer while preserving a substantial charge-transfer character of the lowest excited state.

Structural analysis showed that increasing electron-with-drawing character generally leads to larger NacNac^Me^ bite angles, resulting in ground-state coordination geometries slightly closer to the ideal tetrahedral arrangement around the Cu(I) center. Because only ground-state geometries were optimized, no direct conclusion can be drawn regarding structural rigidity or the suppression of excited-state flattening distortions.

The LED energy decomposition provided complementary insight into metal-ligand interactions. Electron-donating substituents yielded more stabilizing total interaction energies, indicating stronger ground-state covalent bonding, whereas electron-withdrawing groups increased exchange stabilization and orbital overlap. These effects, although not directly correlated one-to-one with the computed spectral shifts, reveal that individual LED components provide complementary information to frontier orbital analysis, capturing subtle variations in metal–ligand covalency induced by ligand substitution.

Overall, this work provides a comprehensive structure–property relationship for a family of heteroleptic Cu(I) complexes by correlating substituent electronics with molecular structure, frontier-orbital energetics, metal–ligand interactions, excited-state character, and charge-transfer absorption properties. These results demonstrate that ligand substitution represents a rational strategy for tuning the electronic and photophysical properties of Cu(I) photosensitizers while highlighting the importance of considering both ground- and excited-state descriptors for their molecular design. Excited-state dynamics of these systems are being investigated, and they will help uncover their suitability for photocatalytic and solar-energy-conversion applications.

## Supplementary information

Below is the link to the electronic supplementary material.

## Supplementary Information

Below is the link to the electronic supplementary material.

Supplementary file 1 (pdf 26494 KB)

## Data Availability

All data supporting the findings of this study are available within the paper and its Supplementary Information.

## Materials availability

Data are provided as Supplementary Information.
