# Supplementary material for: Structure and photophysical properties of copper(I) photosensitizers: a computational study of substituent effects
Source: J Mol Model. 2026 Nov 7;32(12):461. doi: 10.1007/s00894-026-07025-7 (PMC13743570; doi:10.1007/s00894-026-07025-7)
Supplement: Supplementary file 1 [file 894_2026_7025_MOESM1_ESM.pdf]

# Supplementary Information: Structure and Photophysical Properties of Copper(I) Photosensitizers: A Computational Study of Substituent Effects.*

Bruno Campana Severino[1†], Vania Martins Ramos[1†], Ana Paula de Lima Batista[1*]

[1]Departamento de Química, Grupo Computacional de Catálise e Espectroscopia (GCCE), Universidade Federal de São Carlos (UFSCar), Rod. Washington Luís, São Carlos, 13565-905, SP, Brazil.

*Corresponding author(s). E-mail(s): aplbatista@ufscar.br;
Contributing authors: brunocampana@estudante.ufscar.br;
vania.ramos@alumni.usp.br;
†These authors contributed equally to this work.

*Manuscript prepared for JMM Topical Collection XXIII SBQT 2025 September 15, 2026.

**Table S1**: HOMO and LUMO energies and the corresponding HOMO–LUMO energy gap calculated at the DFT/CAM-B3LYP-D3(BJ)/def2-TZVP/C-PCM(toluene) level of theory.

| | HOMO (eV) | LUMO (eV) | HOMO–LUMO gap (eV) |
|---|---|---|---|
| $R = NH_2$ | -5.54 | -0.98 | 4.56 |
| $R = OH$ | -5.62 | -1.15 | 4.47 |
| $R = CH_3$ | -5.62 | -1.19 | 4.43 |
| $R = H$ | -5.66 | -1.32 | 4.34 |
| $R = Cl$ | -5.75 | -1.50 | 4.25 |
| $R = COOCH_3$ | -5.74 | -1.71 | 4.03 |
| $R = CF_3$ | -5.78 | -1.68 | 4.10 |

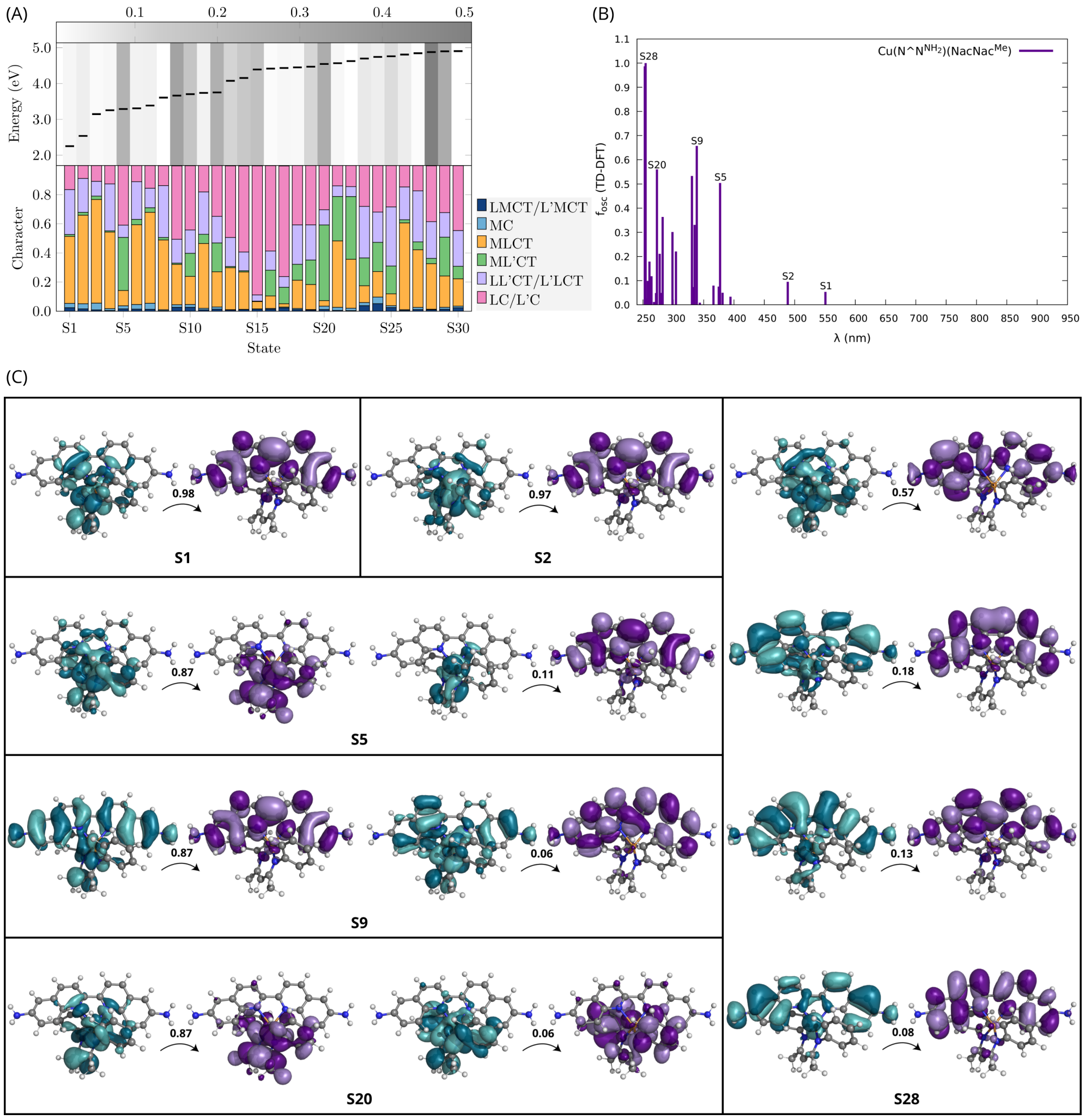


**Fig. S1**: Cu(N^N$^{NH_2}$)(NacNac$^{Me}$) data: (A) Energies and charge-transfer number analysis of the 30 lowest-energy electronic transitions calculated at the TD-DFT/CAM-B3LYP/def2-TZVP/C-PCM(toluene) level of theory using Löwdin population analysis, with the complex fragmented into the copper center (M), the N^N$^{NH_2}$ ligand (L), and the NacNac$^{Me}$ ligand (L'). The intensity of the grayscale shading in the upper panel is proportional to the oscillator strength ($f$) of each transition. (B) Electronic absorption spectrum; and (C) NTO pairs for selected transitions, with the weights indicated above the arrows. The labels below each set of NTOs indicate the corresponding excited states.

**Table S2**: Calculated vertical excitation energies ($E_{\text{exc.}}$), corresponding absorption wavelengths ($\lambda$), oscillator strengths ($f_{\text{osc.}}$), excited-state character contributions, charge-transfer numbers ($q_{\text{CT}}$), and root-mean-square electron–hole separation distances ($d_{eh}$) for the Cu(N^N$^{NH_2}$)(NacNac$^{Me}$) complex. The excited states were computed at the TD-DFT/CAM-B3LYP/def2-TZVP/C-PCM(toluene) level of theory. The excited-state character contributions were obtained from TheoDORE charge-transfer number analysis using Löwdin population analysis, with the complex partitioned into the copper center (M), the N^N$^{NH_2}$ ligand (L), and the NacNac$^{Me}$ ligand (L′). The $q_{\text{CT}}$ values correspond to the TheoDORE charge-transfer numbers, while $d_{eh}$ denotes the root-mean-square electron–hole separation.

| State | $\lambda$ (nm) | $E_{\text{exc.}}$ (eV) | $f_{\text{osc.}}$ | $q_{\text{CT}}$ | $d_{eh}$ (Å) | TheoDORE character contributions | | | | | |
|---|---|---|---|---|---|---|---|---|---|---|---|
| | | | | | | LMCT/L′MCT | MC | MLCT | ML′CT | LL′CT/L′LCT | LC/L′C |
| S1 | 550.7 | 2.25 | 0.027 | 0.806 | 4.221 | 2.4 % | 3.0 % | 47.3 % | 1.3 % | 30.9 % | 16.5 % |
| S2 | 488.7 | 2.54 | 0.048 | 0.876 | 4.076 | 1.6 % | 3.5 % | 62.8 % | 2.0 % | 23.3 % | 8.8 % |
| S3 | 394.1 | 3.14 | 0.017 | 0.845 | 3.750 | 0.9 % | 4.6 % | 73.5 % | 2.4 % | 10.1 % | 10.8 % |
| S4 | 381.0 | 3.25 | 0.025 | 0.861 | 4.575 | 0.5 % | 1.3 % | 53.4 % | 0.9 % | 32.2 % | 12.6 % |
| S5 | 377.1 | 3.29 | 0.256 | 0.569 | 3.713 | 1.6 % | 2.1 % | 47.0 % | 36.4 % | 8.4 % | 40.9 % |
| S6 | 374.9 | 3.31 | 0.038 | 0.858 | 4.237 | 1.4 % | 3.2 % | 58.4 % | 3.7 % | 25.9 % | 11.1 % |
| S7 | 366.2 | 3.38 | 0.040 | 0.803 | 3.994 | 1.3 % | 4.1 % | 65.6 % | 3.1 % | 13.4 % | 15.5 % |
| S8 | 343.8 | 3.61 | 0.004 | 0.855 | 4.775 | 0.4 % | 0.7 % | 49.6 % | 1.7 % | 35.6 % | 13.7 % |
| S9 | 338.6 | 3.66 | 0.334 | 0.476 | 4.433 | 2.7 % | 1.8 % | 28.6 % | 0.8 % | 16.4 % | 50.4 % |
| S10 | 334.9 | 3.70 | 0.168 | 0.538 | 4.054 | 2.7 % | 1.9 % | 35.1 % | 1.59 % | 16.0 % | 44.1 % |
| S11 | 331.7 | 3.74 | 0.037 | 0.808 | 4.538 | 0.9 % | 1.0 % | 50.9 % | 0.9 % | 29.1 % | 18.1 % |
| S12 | 330.5 | 3.75 | 0.271 | 0.637 | 4.042 | 1.4 % | 1.5 % | 43.9 % | 1.98 % | 18.3 % | 34.8 % |
| S13 | 304.3 | 4.07 | 0.112 | 0.504 | 4.302 | 0.8 % | 0.2 % | 29.6 % | 0.8 % | 19.9 % | 49.2 % |
| S14 | 298.5 | 4.15 | 0.153 | 0.405 | 4.126 | 1.1 % | 0.2 % | 26.5 % | 0.8 % | 12.9 % | 59.1 % |
| S15 | 282.4 | 4.39 | 0.185 | 0.110 | 4.253 | 1.4 % | 0.1 % | 5.3 % | 0.3 % | 4.3 % | 88.6 % |
| S16 | 280.7 | 4.42 | 0.017 | 0.408 | 4.462 | 1.4 % | 0.5 % | 26.2 % | 1.4 % | 13.2 % | 58.6 % |
| S17 | 279.7 | 4.43 | 0.025 | 0.232 | 4.555 | 2.3 % | 0.6 % | 13.5 % | 2.3 % | 7.4 % | 76.0 % |
| S18 | 278.6 | 4.45 | 0.024 | 0.585 | 4.708 | 1.1 % | 0.8 % | 30.3 % | 1.1 % | 27.1 % | 40.7 % |
| S19 | 277.4 | 4.47 | 0.107 | 0.589 | 4.540 | 0.8 % | 0.4 % | 34.0 % | 0.8 % | 24.1 % | 40.7 % |
| S20 | 272.8 | 4.54 | 0.285 | 0.674 | 3.613 | 1.2 % | 2.3 % | 55.8 % | 1.2 % | 10.4 % | 30.4 % |
| S21 | 271.5 | 4.57 | 0.025 | 0.840 | 3.871 | 0.5 % | 2.1 % | 76.1 % | 0.5 % | 7.3 % | 13.9 % |
| S22 | 268.0 | 4.62 | 0.006 | 0.839 | 3.749 | 0.4 % | 1.6 % | 76.7 % | 0.4 % | 6.9 % | 14.4 % |
| S23 | 263.9 | 4.70 | 0.060 | 0.698 | 4.547 | 3.8 % | 2.1 % | 30.7 % | 3.8 % | 35.4 % | 28.0 % |
| S24 | 261.4 | 4.74 | 0.047 | 0.636 | 4.223 | 5.2 % | 4.5 % | 37.6 % | 5.2 % | 20.8 % | 31.9 % |
| S25 | 260.5 | 4.76 | 0.091 | 0.703 | 4.685 | 2.5 % | 1.3 % | 27.2 % | 2.5 % | 40.6 % | 28.3 % |
| S26 | 257.9 | 4.81 | 0.050 | 0.848 | 4.508 | 0.6 % | 0.5 % | 61.7 % | 0.6 % | 22.5 % | 14.6 % |
| S27 | 255.9 | 4.84 | 0.013 | 0.812 | 4.793 | 1.1 % | 1.5 % | 45.0 % | 1.1 % | 35.1 % | 17.3 % |
| S28 | 254.2 | 4.88 | 0.508 | 0.611 | 4.488 | 1.0 % | 0.4 % | 34.9 % | 1.0 % | 25.2 % | 38.4 % |
| S29 | 253.1 | 4.90 | 0.291 | 0.664 | 4.296 | 1.4 % | 1.3 % | 48.2 % | 1.4 % | 16.8 % | 32.2 % |
| S30 | 252.8 | 4.90 | 0.502 | 0.542 | 4.443 | 2.4 % | 1.1 % | 27.4 % | 2.4 % | 24.5 % | 44.6 % |

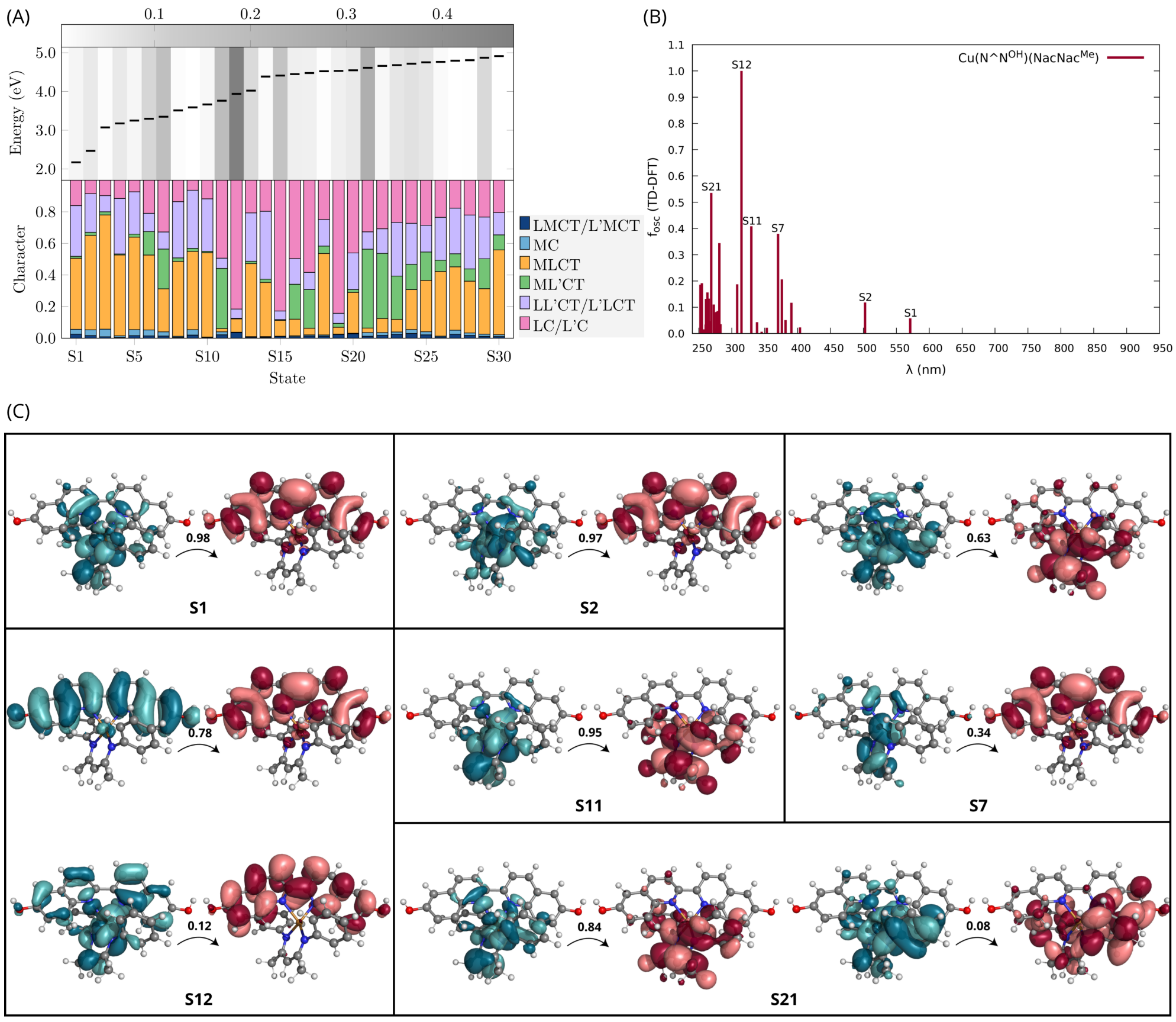


**Fig. S2**: Cu(N^N$^{OH}$)(NacNac$^{Me}$) data: (A) Energies and charge-transfer number analysis of the 30 lowest-energy electronic transitions calculated at the TD-DFT/CAM-B3LYP/def2-TZVP/C-PCM(toluene) level of theory using Löwdin population analysis, with the complex fragmented into the copper center (M), the N^N$^{OH}$ ligand (L), and the NacNac$^{Me}$ ligand (L'). The intensity of the grayscale shading in the upper panel is proportional to the oscillator strength ($f$) of each transition. (B) Electronic absorption spectrum; and (C) NTO pairs for selected transitions, with the weights indicated above the arrows. The labels below each set of NTOs indicate the corresponding excited states.

**Table S3**: Calculated vertical excitation energies ($E_{\text{exc.}}$), corresponding absorption wavelengths ($\lambda$), oscillator strengths ($f_{\text{osc.}}$), excited-state character contributions, charge-transfer numbers ($q_{\text{CT}}$), and root-mean-square electron–hole separation distances ($d_{eh}$) for the Cu(N^N$^{\text{OH}}$)(NacNac$^{\text{Me}}$) complex. The excited states were computed at the TD-DFT/CAM-B3LYP/def2-TZVP/C-PCM(toluene) level of theory. The excited-state character contributions were obtained from TheoDORE charge-transfer number analysis using Löwdin population analysis, with the complex partitioned into the copper center (M), the N^N$^{\text{OH}}$ ligand (L), and the NacNac$^{\text{Me}}$ ligand (L′). The $q_{\text{CT}}$ values correspond to the TheoDORE charge-transfer numbers, while $d_{eh}$ denotes the root-mean-square electron–hole separation.

| State | $\lambda$ (nm) | $E_{\text{exc.}}$ (eV) | $f_{\text{osc.}}$ | $q_{\text{CT}}$ | $d_{eh}$ (Å) | TheoDORE character contributions | | | | | |
|---|---|---|---|---|---|---|---|---|---|---|---|
| | | | | | | LMCT/L′MCT | MC | MLCT | ML′CT | LL′CT/L′LCT | LC/L′C |
| S1 | 571.1 | 2.17 | 0.027 | 0.809 | 4.222 | 2.6 % | 3.0 % | 45.0 % | 1.2 % | 32.1 % | 16.1 % |
| S2 | 502.5 | 2.47 | 0.056 | 0.878 | 4.078 | 1.7 % | 3.6 % | 59.8 % | 1.9 % | 24.5 % | 8.5 % |
| S3 | 403.6 | 3.07 | 0.011 | 0.854 | 3.731 | 0.9 % | 4.8 % | 72.3 % | 2.0 % | 10.3 % | 9.7 % |
| S4 | 390.5 | 3.17 | 0.055 | 0.874 | 4.638 | 0.4 % | 1.1 % | 51.2 % | 0.7 % | 35.1 % | 11.5 % |
| S5 | 381.6 | 3.25 | 0.024 | 0.889 | 4.210 | 1.6 % | 3.8 % | 58.5 % | 1.9 % | 26.8 % | 7.3 % |
| S6 | 376.2 | 3.30 | 0.098 | 0.753 | 3.809 | 1.4 % | 3.8 % | 47.4 % | 14.9 % | 11.5 % | 20.9 % |
| S7 | 370.3 | 3.35 | 0.180 | 0.645 | 3.798 | 1.4 % | 2.7 % | 27.2 % | 25.2 % | 10.8 % | 32.8 % |
| S8 | 353.1 | 3.51 | 0.010 | 0.856 | 4.741 | 0.4 % | 0.8 % | 47.4 % | 2.2 % | 35.6 % | 13.6 % |
| S9 | 345.5 | 3.59 | 0.003 | 0.902 | 4.409 | 2.0 % | 3.4 % | 49.5 % | 1.9 % | 36.8 % | 6.3 % |
| S10 | 338.3 | 3.66 | 0.020 | 0.878 | 4.703 | 0.3 % | 0.4 % | 53.5 % | 0.8 % | 33.2 % | 11.8 % |
| S11 | 329.6 | 3.76 | 0.194 | 0.488 | 3.430 | 2.2 % | 1.9 % | 2.0 % | 38.1 % | 6.5 % | 49.4 % |
| S12 | 314.8 | 3.94 | 0.474 | 0.182 | 4.282 | 3.6 % | 0.2 % | 8.4 % | 0.4 % | 5.9 % | 81.6 % |
| S13 | 308.2 | 4.02 | 0.089 | 0.790 | 4.712 | 0.6 % | 0.3 % | 46.3 % | 1.5 % | 30.6 % | 20.7 % |
| S14 | 282.7 | 4.39 | 0.017 | 0.799 | 4.831 | 0.5 % | 0.5 % | 34.3 % | 2.0 % | 43.0 % | 19.7 % |
| S15 | 281.1 | 4.41 | 0.163 | 0.171 | 3.603 | 1.1 % | 0.1 % | 10.0 % | 0.5 % | 5.5 % | 82.8 % |
| S16 | 278.7 | 4.45 | 0.040 | 0.498 | 4.338 | 0.9 % | 0.6 % | 10.5 % | 22.1 % | 16.3 % | 49.6 % |
| S17 | 276.9 | 4.48 | 0.039 | 0.409 | 4.365 | 1.3 % | 0.8 % | 4.2 % | 24.5 % | 10.9 % | 58.3 % |
| S18 | 274.1 | 4.52 | 0.009 | 0.744 | 4.255 | 1.3 % | 0.8 % | 51.5 % | 4.7 % | 16.9 % | 24.9 % |
| S19 | 273.6 | 4.53 | 0.038 | 0.155 | 4.095 | 2.3 % | 0.3 % | 4.3 % | 2.5 % | 6.4 % | 84.2 % |
| S20 | 272.7 | 4.55 | 0.052 | 0.536 | 4.496 | 2.7 % | 0.4 % | 25.8 % | 1.8 % | 23.1 % | 46.1 % |
| S21 | 268.9 | 4.61 | 0.254 | 0.649 | 3.625 | 1.2 % | 2.3 % | 2.8 % | 49.9 % | 10.9 % | 32.7 % |
| S22 | 266.0 | 4.66 | 0.022 | 0.675 | 3.958 | 1.8 % | 1.8 % | 8.9 % | 41.2 % | 15.6 % | 30.8 % |
| S23 | 264.9 | 4.68 | 0.063 | 0.719 | 4.385 | 2.5 % | 1.4 % | 8.0 % | 27.4 % | 34.0 % | 26.7 % |
| S24 | 262.8 | 4.72 | 0.074 | 0.703 | 4.433 | 3.0 % | 2.5 % | 25.3 % | 16.0 % | 26.0 % | 27.3 % |
| S25 | 260.8 | 4.75 | 0.062 | 0.695 | 4.316 | 2.1 % | 2.0 % | 32.4 % | 18.0 % | 16.9 % | 28.5 % |
| S26 | 260.2 | 4.76 | 0.016 | 0.760 | 4.627 | 0.7 % | 0.5 % | 40.9 % | 7.2 % | 27.2 % | 23.5 % |
| S27 | 258.5 | 4.80 | 0.003 | 0.798 | 4.615 | 2.5 % | 2.5 % | 40.1 % | 8.3 % | 28.9 % | 17.7 % |
| S28 | 257.9 | 4.81 | 0.007 | 0.765 | 4.681 | 1.8 % | 1.5 % | 32.8 % | 7.8 % | 34.1 % | 22.0 % |
| S29 | 254.5 | 4.87 | 0.091 | 0.753 | 4.436 | 1.2 % | 1.4 % | 28.8 % | 18.9 % | 26.4 % | 23.3 % |
| S30 | 252.3 | 4.91 | 0.088 | 0.783 | 4.315 | 1.0 % | 1.2 % | 53.7 % | 9.5 % | 14.1 % | 20.5 % |

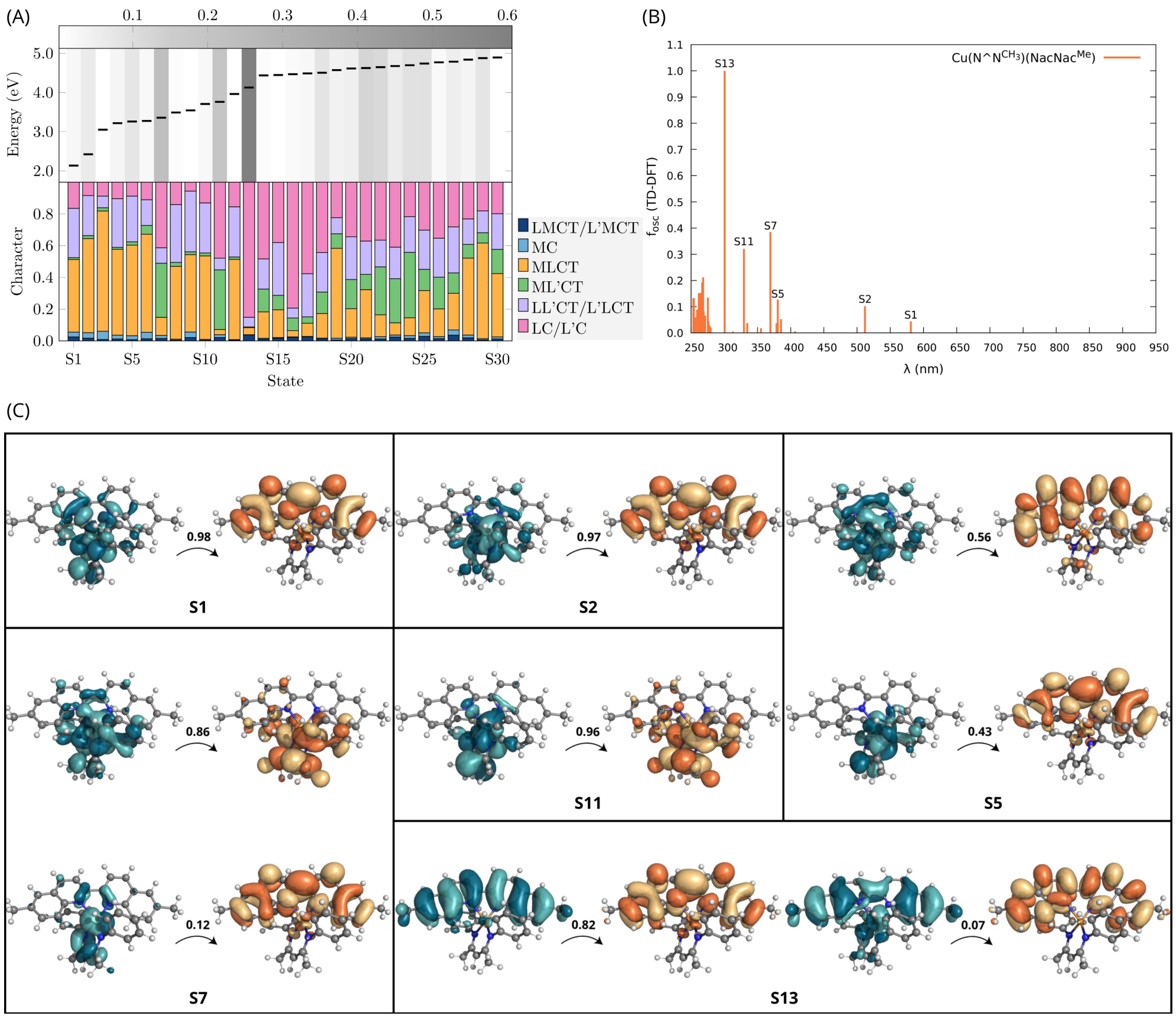


**Fig. S3**: Cu(N^N$^{\mathrm{CH_3}}$)(NacNac$^{\mathrm{Me}}$) data: (A) Energies and charge-transfer number analysis of the 30 lowest-energy electronic transitions calculated at the TD-DFT/CAM-B3LYP/def2-TZVP/C-PCM(toluene) level of theory using Löwdin population analysis, with the complex fragmented into the copper center (M), the N^N$^{\mathrm{CH_3}}$ ligand (L), and the NacNac$^{\mathrm{Me}}$ ligand (L'). The intensity of the grayscale shading in the upper panel is proportional to the oscillator strength ($f$) of each transition. (B) Electronic absorption spectrum; and (C) NTO pairs for selected transitions, with the weights indicated above the arrows. The labels below each set of NTOs indicate the corresponding excited states.

**Table S4**: Calculated vertical excitation energies ($E_{\text{exc.}}$), corresponding absorption wavelengths ($\lambda$), oscillator strengths ($f_{\text{osc.}}$), excited-state character contributions, charge-transfer numbers ($q_{\text{CT}}$), and root-mean-square electron–hole separation distances ($d_{eh}$) for the Cu(N^N$^{\text{CH}_3}$)(NacNac$^{\text{Me}}$) complex. The excited states were computed at the TD-DFT/CAM-B3LYP/def2-TZVP/C-PCM(toluene) level of theory. The excited-state character contributions were obtained from TheoDORE charge-transfer number analysis using Löwdin population analysis, with the complex partitioned into the copper center (M), the N^N$^{\text{CH}_3}$ ligand (L), and the NacNac$^{\text{Me}}$ ligand (L′). The $q_{\text{CT}}$ values correspond to the TheoDORE charge-transfer numbers, while $d_{eh}$ denotes the root-mean-square electron–hole separation.

| State | $\lambda$ (nm) | $E_{\text{exc.}}$ (eV) | $f_{\text{osc.}}$ | $q_{\text{CT}}$ | $d_{eh}$ (Å) | TheoDORE character contributions | | | | | |
|---|---|---|---|---|---|---|---|---|---|---|---|
| | | | | | | LMCT/L′MCT | MC | MLCT | ML′CT | LL′CT/L′LCT | LC/L′C |
| S1 | 581.0 | 2.13 | 0.026 | 0.805 | 4.225 | 2.5 % | 3.0 % | 45.7 % | 1.2 % | 31.1 % | 16.5 % |
| S2 | 512.0 | 2.42 | 0.062 | 0.881 | 4.122 | 1.7 % | 3.5 % | 59.3 % | 1.9 % | 25.2 % | 8.4 % |
| S3 | 406.6 | 3.05 | 0.004 | 0.860 | 3.670 | 0.8 % | 5.2 % | 75.8 % | 2.0 % | 7.3 % | 8.8 % |
| S4 | 385.4 | 3.22 | 0.032 | 0.871 | 4.399 | 1.1 % | 2.4 % | 54.1 % | 1.2 % | 30.7 % | 10.5 % |
| S5 | 380.5 | 3.26 | 0.077 | 0.887 | 4.432 | 0.7 % | 2.5 % | 57.1 % | 2.1 % | 28.8 % | 8.8 % |
| S6 | 378.7 | 3.27 | 0.022 | 0.848 | 3.976 | 1.3 % | 4.1 % | 61.8 % | 5.6 % | 16.2 % | 11.1 % |
| S7 | 369.5 | 3.36 | 0.233 | 0.567 | 3.751 | 1.5 % | 1.9 % | 11.4 % | 34.1 % | 9.8 % | 41.3 % |
| S8 | 355.3 | 3.49 | 0.011 | 0.852 | 4.797 | 0.4 % | 0.6 % | 45.8 % | 2.5 % | 36.4 % | 14.2 % |
| S9 | 349.9 | 3.54 | 0.001 | 0.909 | 4.450 | 2.1 % | 3.5 % | 48.8 % | 1.8 % | 38.3 % | 5.6 % |
| S10 | 334.5 | 3.71 | 0.023 | 0.863 | 4.659 | 0.4 % | 0.6 % | 52.6 % | 1.9 % | 31.5 % | 13.1 % |
| S11 | 329.6 | 3.76 | 0.194 | 0.503 | 3.454 | 2.1 % | 1.8 % | 3.3 % | 37.6 % | 7.4 % | 47.9 % |
| S12 | 313.0 | 3.96 | 0.003 | 0.841 | 4.733 | 0.3 % | 0.4 % | 50.6 % | 1.5 % | 31.6 % | 15.5 % |
| S13 | 300.4 | 4.13 | 0.606 | 0.147 | 4.194 | 3.7 % | 0.1 % | 4.4 % | 0.5 % | 6.0 % | 85.2 % |
| S14 | 279.5 | 4.44 | 0.013 | 0.511 | 4.434 | 1.1 % | 0.5 % | 16.7 % | 14.4 % | 19.0 % | 48.4 % |
| S15 | 278.8 | 4.45 | 0.004 | 0.615 | 4.593 | 1.6 % | 0.5 % | 17.5 % | 9.1 % | 33.3 % | 38.0 % |
| S16 | 277.5 | 4.47 | 0.017 | 0.203 | 4.147 | 2.2 % | 0.3 % | 3.9 % | 8.0 % | 6.2 % | 79.4 % |
| S17 | 276.3 | 4.49 | 0.026 | 0.421 | 4.477 | 2.5 % | 0.2 % | 8.4 % | 4.0 % | 27.2 % | 57.7 % |
| S18 | 275.3 | 4.50 | 0.082 | 0.551 | 4.602 | 1.2 % | 0.6 % | 15.5 % | 13.5 % | 24.9 % | 44.4 % |
| S19 | 271.2 | 4.57 | 0.040 | 0.765 | 4.060 | 0.5 % | 1.1 % | 56.6 % | 9.1 % | 10.1 % | 22.4 % |
| S20 | 268.7 | 4.61 | 0.050 | 0.647 | 4.430 | 1.4 % | 0.8 % | 18.0 % | 18.4 % | 27.0 % | 34.4 % |
| S21 | 268.1 | 4.62 | 0.128 | 0.621 | 4.174 | 1.2 % | 0.7 % | 30.3 % | 9.7 % | 21.0 % | 37.1 % |
| S22 | 266.8 | 4.65 | 0.117 | 0.620 | 4.028 | 1.1 % | 1.5 % | 13.8 % | 30.2 % | 16.9 % | 36.5 % |
| S23 | 265.2 | 4.68 | 0.059 | 0.578 | 4.084 | 2.4 % | 1.3 % | 7.7 % | 27.8 % | 19.9 % | 41.0 % |
| S24 | 264.0 | 4.70 | 0.092 | 0.765 | 4.107 | 1.6 % | 1.8 % | 11.2 % | 41.2 % | 22.5 % | 21.7 % |
| S25 | 261.6 | 4.74 | 0.092 | 0.675 | 4.432 | 2.8 % | 2.2 % | 26.7 % | 13.4 % | 24.7 % | 30.2 % |
| S26 | 259.9 | 4.77 | 0.022 | 0.634 | 4.588 | 1.4 % | 1.3 % | 17.5 % | 20.0 % | 24.6 % | 35.3 % |
| S27 | 259.0 | 4.79 | 0.054 | 0.685 | 4.511 | 3.6 % | 3.3 % | 23.1 % | 12.8 % | 29.0 % | 28.2 % |
| S28 | 256.2 | 4.84 | 0.036 | 0.751 | 4.275 | 1.9 % | 1.8 % | 48.5 % | 8.7 % | 16.0 % | 23.2 % |
| S29 | 254.2 | 4.88 | 0.081 | 0.811 | 4.414 | 0.6 % | 0.8 % | 60.3 % | 6.5 % | 13.7 % | 18.1 % |
| S30 | 253.3 | 4.89 | 0.080 | 0.787 | 4.340 | 1.1 % | 1.4 % | 39.9 % | 15.3 % | 22.4 % | 19.9 % |

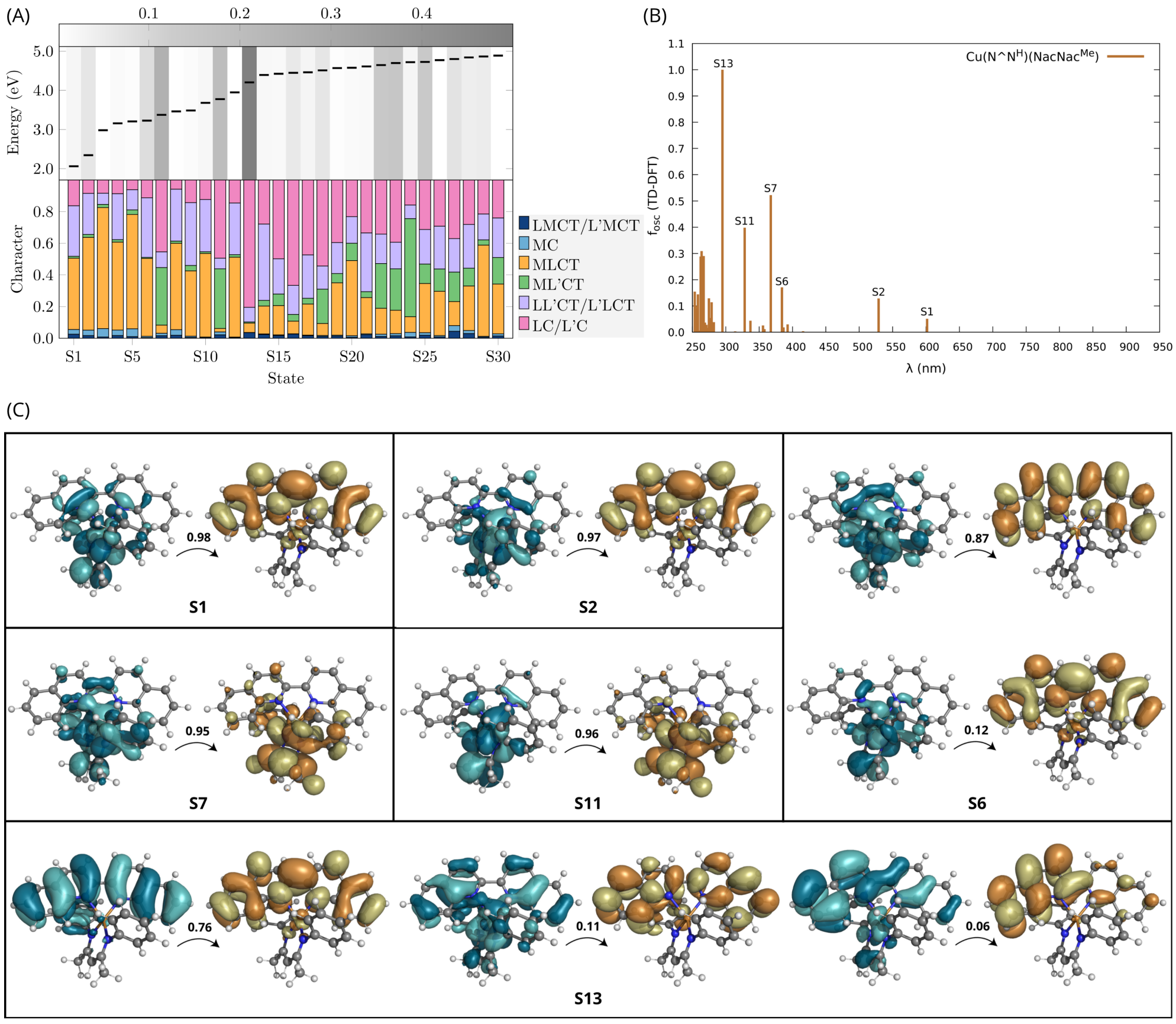


**Fig. S4**: Cu(N^N$^{H}$)(NacNac$^{Me}$) data: (A) Energies and charge-transfer number analysis of the 30 lowest-energy electronic transitions calculated at the TD-DFT/CAM-B3LYP/def2-TZVP/C-PCM(toluene) level of theory using Löwdin population analysis, with the complex fragmented into the copper center (M), the N^N$^{H}$ ligand (L), and the NacNac$^{Me}$ ligand (L'). The intensity of the grayscale shading in the upper panel is proportional to the oscillator strength ($f$) of each transition. (B) Electronic absorption spectrum; and (C) NTO pairs for selected transitions, with the weights indicated above the arrows. The labels below each set of NTOs indicate the corresponding excited states.

**Table S5**: Calculated vertical excitation energies ($E_{\text{exc.}}$), corresponding absorption wavelengths ($\lambda$), oscillator strengths ($f_{\text{osc.}}$), excited-state character contributions, charge-transfer numbers ($q_{\text{CT}}$), and root-mean-square electron–hole separation distances ($d_{eh}$) for the Cu(N^N$^{\text{H}}$)(NacNac$^{\text{Me}}$) complex. The excited states were computed at the TD-DFT/CAM-B3LYP/def2-TZVP/C-PCM(toluene) level of theory. The excited-state character contributions were obtained from TheoDORE charge-transfer number analysis using Löwdin population analysis, with the complex partitioned into the copper center (M), the N^N$^{\text{H}}$ ligand (L), and the NacNac$^{\text{Me}}$ ligand (L′). The $q_{\text{CT}}$ values correspond to the TheoDORE charge-transfer numbers, while $d_{eh}$ denotes the root-mean-square electron–hole separation.

| State | $\lambda$ (nm) | $E_{\text{exc.}}$ (eV) | $f_{\text{osc.}}$ | $q_{\text{CT}}$ | $d_{eh}$ (Å) | TheoDORE character contributions | | | | | |
|---|---|---|---|---|---|---|---|---|---|---|---|
| | | | | | | LMCT/L′MCT | MC | MLCT | ML′CT | LL′CT/L′LCT | LC/L′C |
| S1 | 601.7 | 2.06 | 0.025 | 0.807 | 4.220 | 2.5 % | 3.0 % | 45.1 % | 1.2 % | 31.9 % | 16.3 % |
| S2 | 529.0 | 2.34 | 0.064 | 0.881 | 4.118 | 1.7 % | 3.4 % | 58.6 % | 1.8 % | 26.0 % | 8.5 % |
| S3 | 415.7 | 2.98 | 0.002 | 0.863 | 3.642 | 0.8 % | 5.3 % | 76.5 % | 2.0 % | 7.1 % | 8.4 % |
| S4 | 392.6 | 3.16 | 0.015 | 0.878 | 4.247 | 1.7 % | 3.5 % | 55.5 % | 1.6 % | 29.0 % | 8.7 % |
| S5 | 386.6 | 3.21 | 0.009 | 0.889 | 3.848 | 1.0 % | 5.0 % | 72.2 % | 2.9 % | 12.8 % | 6.2 % |
| S6 | 384.2 | 3.23 | 0.085 | 0.879 | 4.733 | 0.4 % | 0.8 % | 49.2 % | 0.6 % | 37.6 % | 11.3 % |
| S7 | 367.4 | 3.38 | 0.261 | 0.530 | 3.747 | 1.6 % | 1.6 % | 5.1 % | 36.4 % | 9.9 % | 45.4 % |
| S8 | 358.0 | 3.46 | 0.006 | 0.906 | 4.344 | 1.9 % | 3.5 % | 54.6 % | 1.4 % | 32.8 % | 5.8 % |
| S9 | 355.7 | 3.49 | 0.013 | 0.849 | 4.782 | 0.5 % | 0.8 % | 41.2 % | 3.4 % | 39.7 % | 14.3 % |
| S10 | 336.9 | 3.68 | 0.022 | 0.872 | 4.709 | 0.3 % | 0.5 % | 52.8 % | 1.1 % | 33.0 % | 12.4 % |
| S11 | 328.3 | 3.78 | 0.199 | 0.488 | 3.418 | 2.2 % | 1.8 % | 2.2 % | 37.7 % | 6.7 % | 49.4 % |
| S12 | 314.0 | 3.95 | 0.002 | 0.850 | 4.701 | 0.3 % | 0.4 % | 50.4 % | 1.6 % | 32.6 % | 14.6 % |
| S13 | 294.9 | 4.20 | 0.500 | 0.195 | 4.061 | 3.6 % | 0.1 % | 5.9 % | 0.7 % | 9.2 % | 80.5 % |
| S14 | 282.3 | 4.39 | 0.019 | 0.717 | 4.678 | 2.4 % | 0.4 % | 17.6 % | 3.5 % | 48.2 % | 27.9 % |
| S15 | 280.3 | 4.42 | 0.017 | 0.497 | 4.455 | 1.7 % | 0.5 % | 18.5 % | 7.3 % | 22.3 % | 49.8 % |
| S16 | 278.7 | 4.45 | 0.057 | 0.333 | 4.347 | 2.7 % | 0.1 % | 8.0 % | 4.3 % | 18.3 % | 66.6 % |
| S17 | 277.9 | 4.46 | 0.027 | 0.523 | 4.595 | 1.6 % | 0.3 % | 19.6 % | 3.7 % | 27.4 % | 47.4 % |
| S18 | 274.9 | 4.51 | 0.064 | 0.450 | 4.453 | 1.2 % | 0.6 % | 7.6 % | 21.7 % | 14.6 % | 54.4 % |
| S19 | 271.4 | 4.57 | 0.008 | 0.599 | 4.378 | 1.4 % | 0.5 % | 33.1 % | 5.9 % | 19.6 % | 39.5 % |
| S20 | 270.9 | 4.58 | 0.014 | 0.758 | 4.321 | 0.6 % | 1.0 % | 47.5 % | 11.0 % | 16.8 % | 23.2 % |
| S21 | 268.9 | 4.61 | 0.018 | 0.660 | 4.706 | 2.3 % | 0.5 % | 22.9 % | 3.8 % | 37.1 % | 33.5 % |
| S22 | 266.9 | 4.65 | 0.145 | 0.644 | 4.041 | 1.2 % | 1.4 % | 16.4 % | 28.2 % | 18.6 % | 34.2 % |
| S23 | 263.9 | 4.70 | 0.154 | 0.593 | 3.958 | 1.6 % | 1.4 % | 14.8 % | 26.2 % | 16.8 % | 39.4 % |
| S24 | 262.7 | 4.72 | 0.026 | 0.813 | 3.516 | 0.9 % | 2.9 % | 9.9 % | 61.9 % | 8.6 % | 15.8 % |
| S25 | 262.4 | 4.73 | 0.144 | 0.671 | 4.314 | 1.9 % | 1.6 % | 31.1 % | 12.3 % | 21.8 % | 31.3 % |
| S26 | 259.8 | 4.77 | 0.003 | 0.700 | 4.619 | 0.9 % | 0.8 % | 27.9 % | 14.1 % | 27.0 % | 29.2 % |
| S27 | 258.3 | 4.80 | 0.072 | 0.593 | 4.275 | 4.4 % | 3.6 % | 15.2 % | 18.6 % | 21.2 % | 37.0 % |
| S28 | 256.1 | 4.84 | 0.044 | 0.700 | 4.325 | 3.0 % | 1.9 % | 28.1 % | 11.3 % | 27.6 % | 28.1 % |
| S29 | 254.9 | 4.86 | 0.050 | 0.779 | 4.339 | 0.7 % | 0.6 % | 57.6 % | 3.3 % | 16.4 % | 21.5 % |
| S30 | 253.7 | 4.89 | 0.077 | 0.746 | 4.338 | 1.4 % | 1.4 % | 31.5 % | 16.7 % | 25.0 % | 24.0 % |

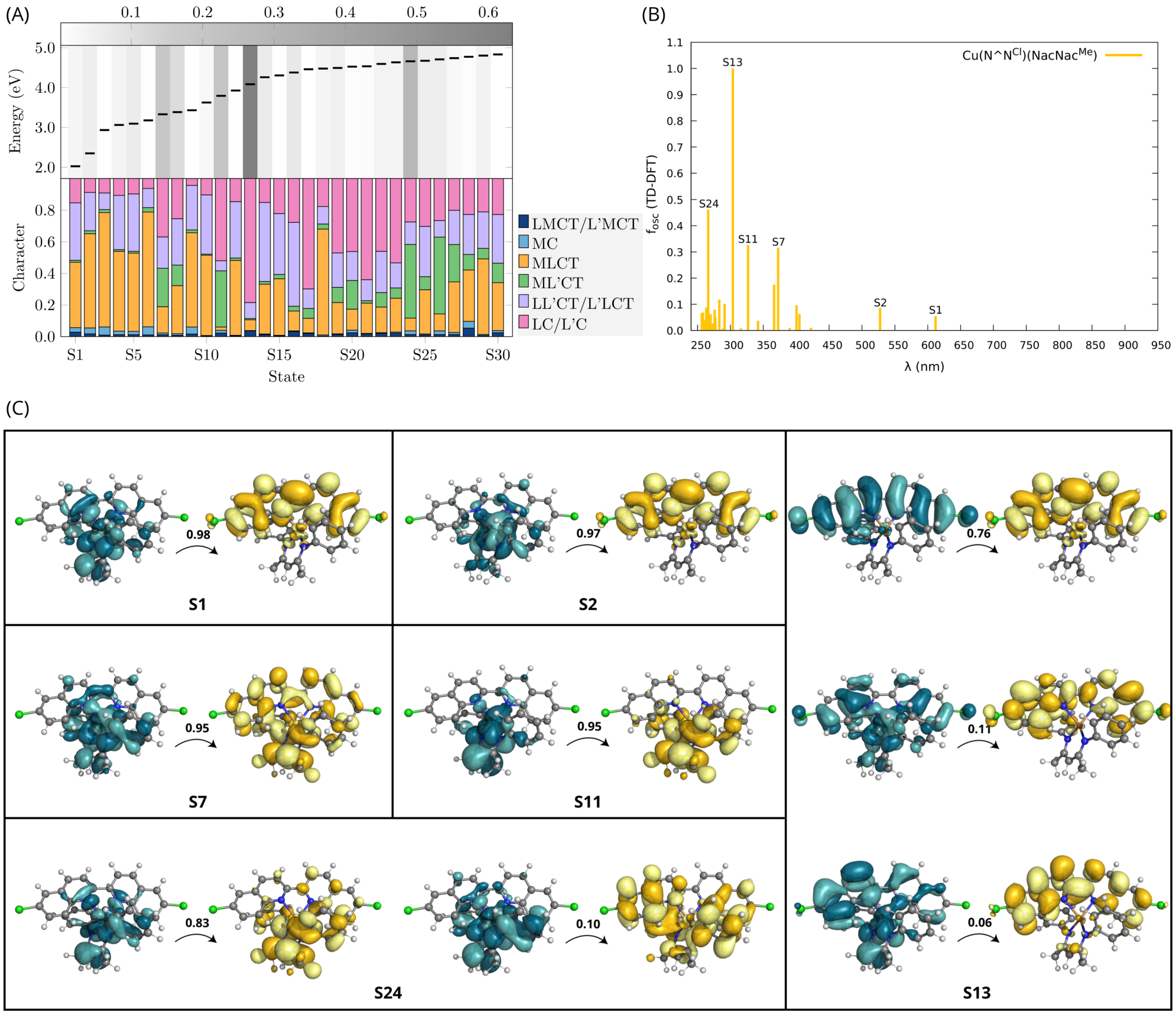


**Fig. S5**: Cu(N^N$^{Cl}$)(NacNac$^{Me}$) data: (A) Energies and charge-transfer number analysis of the 30 lowest-energy electronic transitions calculated at the TD-DFT/CAM-B3LYP/def2-TZVP/C-PCM(toluene) level of theory using Löwdin population analysis, with the complex fragmented into the copper center (M), the N^N$^{Cl}$ ligand (L), and the NacNac$^{Me}$ ligand (L'). The intensity of the grayscale shading in the upper panel is proportional to the oscillator strength ($f$) of each transition. (B) Electronic absorption spectrum; and (C) NTO pairs for selected transitions, with the weights indicated above the arrows. The labels below each set of NTOs indicate the corresponding excited states.

**Table S6**: Calculated vertical excitation energies ($E_{\text{exc.}}$), corresponding absorption wavelengths ($\lambda$), oscillator strengths ($f_{\text{osc.}}$), excited-state character contributions, charge-transfer numbers ($q_{\text{CT}}$), and root-mean-square electron–hole separation distances ($d_{eh}$) for the Cu(N^N$^{\text{Cl}}$)(NacNac$^{\text{Me}}$) complex. The excited states were computed at the TD-DFT/CAM-B3LYP/def2-TZVP/C-PCM(toluene) level of theory. The excited-state character contributions were obtained from TheoDORE charge-transfer number analysis using Löwdin population analysis, with the complex partitioned into the copper center (M), the N^N$^{\text{Cl}}$ ligand (L), and the NacNac$^{\text{Me}}$ ligand (L′). The $q_{\text{CT}}$ values correspond to the TheoDORE charge-transfer numbers, while $d_{eh}$ denotes the root-mean-square electron–hole separation.

| State | $\lambda$ (nm) | $E_{\text{exc.}}$ (eV) | $f_{\text{osc.}}$ | $q_{\text{CT}}$ | $d_{eh}$ (Å) | TheoDORE character contributions | | | | | |
|---|---|---|---|---|---|---|---|---|---|---|---|
| | | | | | | LMCT/L′MCT | MC | MLCT | ML′CT | LL′CT/L′LCT | LC/L′C |
| S1 | 612.0 | 2.03 | 0.034 | 0.818 | 4.363 | 2.8 % | 2.8 % | 41.4 % | 1.2 % | 36.4 % | 15.4 % |
| S2 | 527.5 | 2.35 | 0.055 | 0.875 | 4.113 | 1.7 % | 3.8 % | 59.6 % | 1.9 % | 24.3 % | 8.7 % |
| S3 | 422.4 | 2.94 | 0.006 | 0.858 | 3.757 | 1.0 % | 5.1 % | 72.4 % | 1.9 % | 10.5 % | 9.1 % |
| S4 | 404.7 | 3.06 | 0.040 | 0.872 | 4.502 | 1.2 % | 2.2 % | 50.5 % | 1.1 % | 34.3 % | 10.6 % |
| S5 | 400.5 | 3.10 | 0.061 | 0.882 | 4.601 | 1.2 % | 2.1 % | 49.5 % | 1.1 % | 36.3 % | 9.7 % |
| S6 | 390.2 | 3.18 | 0.006 | 0.886 | 3.878 | 1.0 % | 5.2 % | 72.7 % | 2.8 % | 12.1 % | 6.3 % |
| S7 | 372.4 | 3.33 | 0.200 | 0.619 | 4.137 | 1.2 % | 1.1 % | 16.6 % | 24.4 % | 19.8 % | 36.9 % |
| S8 | 366.3 | 3.39 | 0.111 | 0.736 | 4.519 | 0.9 % | 1.0 % | 30.4 % | 13.0 % | 29.4 % | 25.4 % |
| S9 | 361.3 | 3.43 | 0.001 | 0.912 | 4.229 | 1.6 % | 4.5 % | 59.6 % | 1.8 % | 28.2 % | 4.3 % |
| S10 | 342.0 | 3.63 | 0.024 | 0.893 | 4.800 | 0.3 % | 0.4 % | 50.8 % | 0.7 % | 37.5 % | 10.3 % |
| S11 | 326.8 | 3.79 | 0.207 | 0.463 | 3.443 | 2.2 % | 1.7 % | 2.1 % | 35.6 % | 6.4 % | 52.0 % |
| S12 | 315.8 | 3.93 | 0.005 | 0.850 | 4.771 | 0.4 % | 0.4 % | 47.4 % | 1.5 % | 35.6 % | 14.6 % |
| S13 | 303.5 | 4.09 | 0.634 | 0.214 | 4.341 | 3.7 % | 0.1 % | 6.8 % | 0.8 % | 10.1 % | 78.5 % |
| S14 | 290.9 | 4.26 | 0.064 | 0.845 | 4.847 | 1.3 % | 0.4 % | 31.5 % | 1.7 % | 50.0 % | 15.1 % |
| S15 | 287.9 | 4.31 | 0.005 | 0.775 | 4.859 | 0.5 % | 0.3 % | 35.8 % | 2.8 % | 38.5 % | 22.2 % |
| S16 | 283.1 | 4.38 | 0.074 | 0.719 | 4.935 | 3.2 % | 0.4 % | 12.6 % | 3.0 % | 53.1 % | 27.7 % |
| S17 | 277.9 | 4.46 | 0.004 | 0.298 | 4.263 | 2.0 % | 0.3 % | 9.2 % | 6.3 % | 12.4 % | 69.8 % |
| S18 | 276.9 | 4.48 | 0.033 | 0.816 | 4.244 | 0.3 % | 0.7 % | 67.0 % | 3.2 % | 11.1 % | 17.7 % |
| S19 | 275.9 | 4.49 | 0.050 | 0.526 | 4.512 | 1.4 % | 0.4 % | 19.7 % | 9.6 % | 21.8 % | 47.1 % |
| S20 | 273.9 | 4.53 | 0.013 | 0.522 | 4.461 | 2.4 % | 1.6 % | 13.3 % | 18.2 % | 18.3 % | 46.2 % |
| S21 | 273.4 | 4.54 | 0.017 | 0.357 | 4.209 | 1.8 % | 0.3 % | 19.2 % | 1.5 % | 13.3 % | 64.0 % |
| S22 | 269.8 | 4.59 | 0.039 | 0.535 | 4.481 | 2.0 % | 0.5 % | 16.2 % | 9.2 % | 26.1 % | 46.1 % |
| S23 | 267.5 | 4.64 | 0.036 | 0.460 | 4.254 | 2.2 % | 0.7 % | 21.4 % | 6.6 % | 15.8 % | 53.3 % |
| S24 | 266.0 | 4.66 | 0.292 | 0.702 | 3.803 | 1.3 % | 2.3 % | 8.1 % | 46.6 % | 14.2 % | 27.5 % |
| S25 | 265.3 | 4.67 | 0.055 | 0.691 | 4.618 | 1.1 % | 0.6 % | 27.9 % | 8.3 % | 31.8 % | 30.3 % |
| S26 | 263.3 | 4.71 | 0.056 | 0.711 | 3.700 | 1.3 % | 2.3 % | 10.7 % | 48.6 % | 10.5 % | 26.6 % |
| S27 | 261.6 | 4.74 | 0.025 | 0.786 | 4.214 | 1.3 % | 1.3 % | 32.0 % | 23.6 % | 21.7 % | 20.1 % |
| S28 | 259.7 | 4.77 | 0.013 | 0.730 | 4.344 | 5.3 % | 4.3 % | 32.6 % | 9.8 % | 25.3 % | 22.7 % |
| S29 | 258.1 | 4.80 | 0.043 | 0.782 | 4.538 | 0.7 % | 0.7 % | 47.8 % | 6.7 % | 23.1 % | 21.1 % |
| S30 | 256.5 | 4.83 | 0.042 | 0.759 | 4.583 | 2.4 % | 1.4 % | 30.3 % | 12.3 % | 30.8 % | 22.7 % |

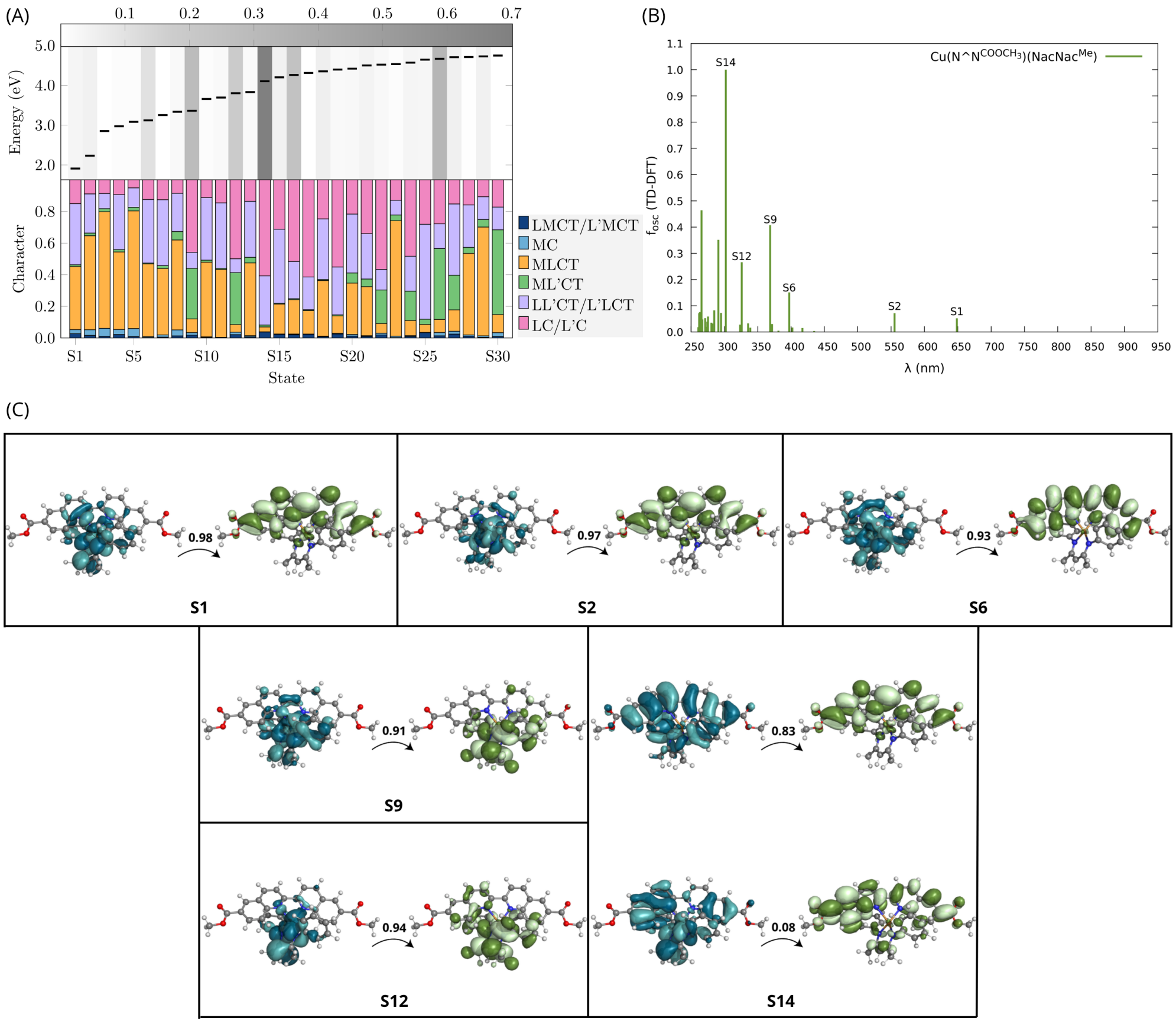


**Fig. S6**: Cu(N^N$^{COOCH_3}$)(NacNac$^{Me}$) data: (A) Energies and charge-transfer number analysis of the 30 lowest-energy electronic transitions calculated at the TD-DFT/CAM-B3LYP/def2-TZVP/C-PCM(toluene) level of theory using Löwdin population analysis, with the complex fragmented into the copper center (M), the N^N$^{COOCH_3}$ ligand (L), and the NacNac$^{Me}$ ligand (L'). The intensity of the grayscale shading in the upper panel is proportional to the oscillator strength ($f$) of each transition. (B) Electronic absorption spectrum; and (C) NTO pairs for selected transitions, with the weights indicated above the arrows. The labels below each set of NTOs indicate the corresponding excited states.

**Table S7**: Calculated vertical excitation energies ($E_{\mathrm{exc.}}$), corresponding absorption wavelengths ($\lambda$), oscillator strengths ($f_{\mathrm{osc.}}$), excited-state character contributions, charge-transfer numbers ($q_{\mathrm{CT}}$), and root-mean-square electron–hole separation distances ($d_{eh}$) for the Cu(N^N$^{\mathrm{COOCH_3}}$)(NacNac$^{\mathrm{Me}}$) complex. The excited states were computed at the TD-DFT/CAM-B3LYP/def2-TZVP/C-PCM(toluene) level of theory. The excited-state character contributions were obtained from TheoDORE charge-transfer number analysis using Löwdin population analysis, with the complex partitioned into the copper center (M), the N^N$^{\mathrm{COOCH_3}}$ ligand (L), and the NacNac$^{\mathrm{Me}}$ ligand (L′). The $q_{\mathrm{CT}}$ values correspond to the TheoDORE charge-transfer numbers, while $d_{eh}$ denotes the root-mean-square electron–hole separation.

| State | $\lambda$ (nm) | $E_{\mathrm{exc.}}$ (eV) | $f_{\mathrm{osc.}}$ | $q_{\mathrm{CT}}$ | $d_{eh}$ (Å) | TheoDORE character contributions | | | | | |
|---|---|---|---|---|---|---|---|---|---|---|---|
| | | | | | | LMCT/L′MCT | MC | MLCT | ML′CT | LL′CT/L′LCT | LC/L′C |
| S1 | 648.7 | 1.91 | 0.037 | 0.823 | 4.606 | 2.7 % | 2.5 % | 39.9 % | 1.1 % | 38.6 % | 15.1 % |
| S2 | 555.2 | 2.23 | 0.050 | 0.876 | 4.319 | 1.6 % | 3.5 % | 59.4 % | 1.7 % | 24.7 % | 8.9 % |
| S3 | 434.7 | 2.85 | 0.002 | 0.862 | 3.887 | 1.0 % | 5.1 % | 73.7 % | 1.9 % | 9.5 % | 8.7 % |
| S4 | 417.1 | 2.97 | 0.011 | 0.875 | 4.581 | 2.2 % | 3.2 % | 49.0 % | 1.5 % | 34.8 % | 9.3 % |
| S5 | 402.1 | 3.08 | 0.012 | 0.898 | 4.027 | 0.9 % | 5.1 % | 74.4 % | 2.3 % | 12.3 % | 5.1 % |
| S6 | 397.3 | 3.12 | 0.106 | 0.870 | 4.920 | 0.3 % | 0.5 % | 46.0 % | 0.5 % | 40.1 % | 12.5 % |
| S7 | 381.0 | 3.25 | 0.003 | 0.860 | 4.931 | 0.6 % | 1.4 % | 42.0 % | 1.7 % | 41.7 % | 12.6 % |
| S8 | 371.6 | 3.34 | 0.021 | 0.877 | 4.308 | 1.3 % | 3.8 % | 56.9 % | 5.4 % | 24.1 % | 8.6 % |
| S9 | 368.8 | 3.36 | 0.286 | 0.525 | 3.843 | 1.8 % | 1.6 % | 8.7 % | 31.9 % | 10.1 % | 45.9 % |
| S10 | 338.9 | 3.66 | 0.012 | 0.884 | 5.061 | 0.3 % | 0.4 % | 47.2 % | 1.3 % | 39.6 % | 11.2 % |
| S11 | 335.6 | 3.69 | 0.023 | 0.853 | 5.340 | 0.3 % | 0.2 % | 42.9 % | 0.7 % | 41.4 % | 14.6 % |
| S12 | 326.1 | 3.80 | 0.186 | 0.485 | 3.558 | 2.1 % | 1.5 % | 4.8 % | 32.8 % | 8.8 % | 49.9 % |
| S13 | 323.7 | 3.83 | 0.019 | 0.857 | 4.963 | 0.7 % | 0.7 % | 46.0 % | 3.5 % | 35.5 % | 13.5 % |
| S14 | 302.1 | 4.10 | 0.701 | 0.391 | 4.690 | 3.7 % | 0.2 % | 3.1 % | 1.2 % | 31.1 % | 60.7 % |
| S15 | 295.1 | 4.20 | 0.051 | 0.686 | 5.097 | 2.3 % | 0.2 % | 18.9 % | 0.5 % | 46.9 % | 31.2 % |
| S16 | 291.0 | 4.26 | 0.246 | 0.479 | 4.848 | 2.1 % | 0.4 % | 21.8 % | 0.6 % | 23.6 % | 51.6 % |
| S17 | 287.6 | 4.31 | 0.000 | 0.384 | 4.769 | 2.3 % | 0.1 % | 14.9 % | 0.5 % | 20.7 % | 61.5 % |
| S18 | 284.9 | 4.35 | 0.058 | 0.749 | 4.943 | 0.8 % | 0.4 % | 35.2 % | 0.7 % | 38.3 % | 24.7 % |
| S19 | 282.0 | 4.40 | 0.023 | 0.444 | 4.746 | 2.5 % | 0.5 % | 11.0 % | 0.6 % | 30.3 % | 55.1 % |
| S20 | 280.5 | 4.42 | 0.025 | 0.774 | 5.041 | 1.2 % | 1.0 % | 32.5 % | 6.4 % | 37.2 % | 21.7 % |
| S21 | 275.5 | 4.50 | 0.041 | 0.656 | 4.787 | 1.0 % | 0.4 % | 31.0 % | 4.8 % | 28.7 % | 34.0 % |
| S22 | 274.2 | 4.52 | 0.027 | 0.422 | 4.419 | 1.9 % | 1.1 % | 6.1 % | 21.2 % | 13.0 % | 56.7 % |
| S23 | 273.4 | 4.53 | 0.013 | 0.862 | 4.305 | 0.2 % | 0.9 % | 73.0 % | 3.6 % | 9.3 % | 13.0 % |
| S24 | 271.2 | 4.57 | 0.037 | 0.512 | 4.493 | 0.9 % | 0.5 % | 9.6 % | 18.6 % | 22.1 % | 48.3 % |
| S25 | 266.9 | 4.65 | 0.034 | 0.717 | 4.800 | 3.4 % | 0.2 % | 5.0 % | 3.3 % | 60.0 % | 28.1 % |
| S26 | 265.6 | 4.67 | 0.325 | 0.701 | 3.852 | 1.3 % | 2.1 % | 8.3 % | 44.8 % | 15.7 % | 27.8 % |
| S27 | 263.4 | 4.71 | 0.053 | 0.829 | 4.833 | 2.4 % | 1.8 % | 13.5 % | 21.9 % | 45.0 % | 15.3 % |
| S28 | 263.1 | 4.71 | 0.004 | 0.834 | 4.621 | 1.2 % | 0.7 % | 51.6 % | 3.8 % | 26.9 % | 15.9 % |
| S29 | 262.2 | 4.73 | 0.050 | 0.884 | 4.562 | 0.4 % | 1.0 % | 68.8 % | 4.8 % | 14.4 % | 10.7 % |
| S30 | 261.1 | 4.75 | 0.013 | 0.803 | 3.777 | 0.9 % | 2.4 % | 11.4 % | 53.6 % | 14.4 % | 17.3 % |

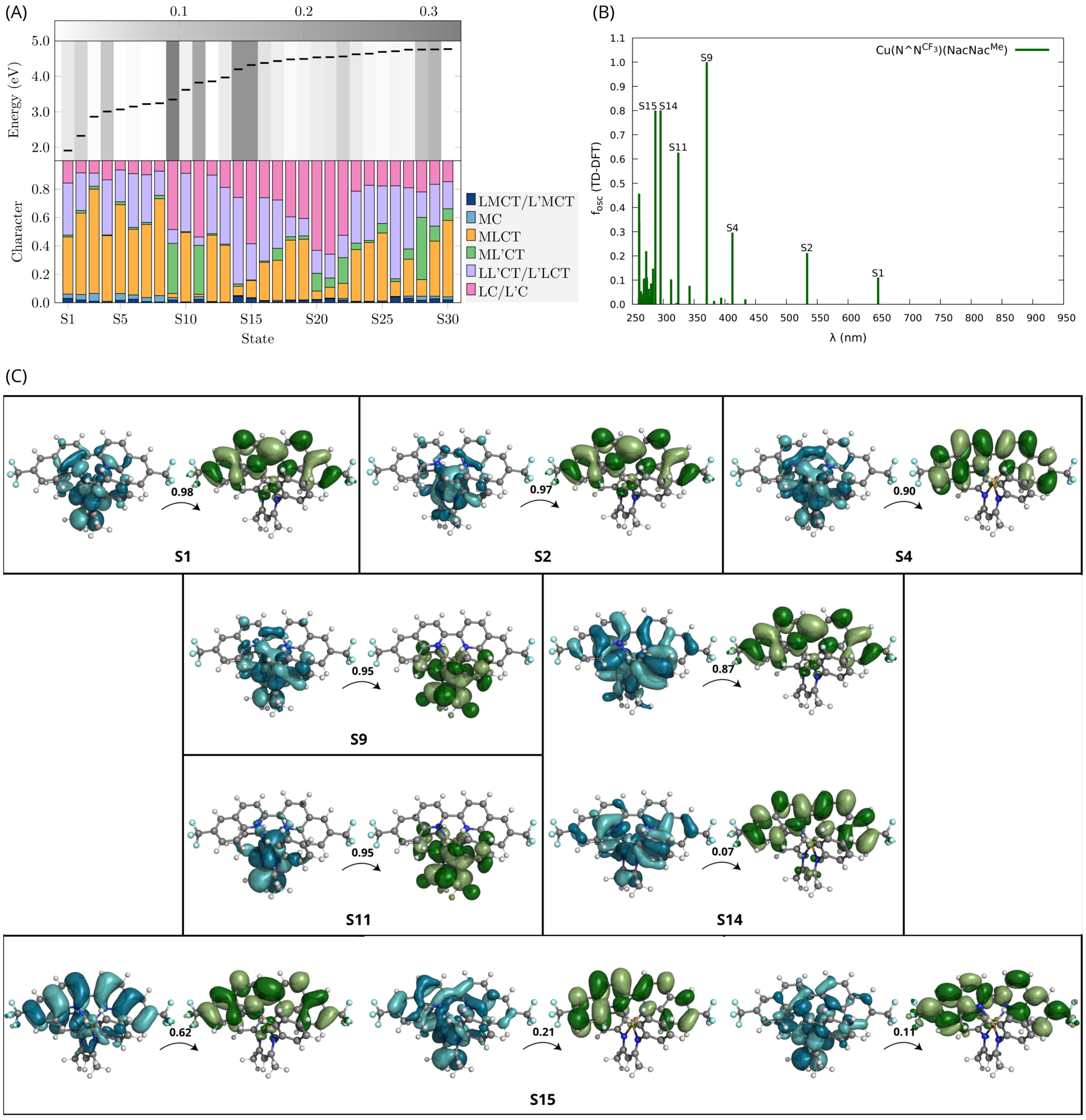


**Fig. S7**: Cu(N^N$^{CF_3}$)(NacNac$^{Me}$) data: (A) Energies and charge-transfer number analysis of the 30 lowest-energy electronic transitions calculated at the TD-DFT/CAM-B3LYP/def2-TZVP/C-PCM(toluene) level of theory using Löwdin population analysis, with the complex fragmented into the copper center (M), the N^N$^{CF_3}$ ligand (L), and the NacNac$^{Me}$ ligand (L'). The intensity of the grayscale shading in the upper panel is proportional to the oscillator strength ($f$) of each transition. (B) Electronic absorption spectrum; and (C) NTO pairs for selected transitions, with the weights indicated above the arrows. The labels below each set of NTOs indicate the corresponding excited states.

**Table S8**: Calculated vertical excitation energies ($E_{\mathrm{exc.}}$), corresponding absorption wavelengths ($\lambda$), oscillator strengths ($f_{\mathrm{osc.}}$), excited-state character contributions, charge-transfer numbers ($q_{\mathrm{CT}}$), and root-mean-square electron–hole separation distances ($d_{eh}$) for the Cu(N^N$^{\mathrm{CF3}}$)(NacNac$^{\mathrm{Me}}$) complex. The excited states were computed at the TD-DFT/CAM-B3LYP/def2-TZVP/C-PCM(toluene) level of theory. The excited-state character contributions were obtained from TheoDORE charge-transfer number analysis using Löwdin population analysis, with the complex partitioned into the copper center (M), the N^N$^{\mathrm{CF3}}$ ligand (L), and the NacNac$^{\mathrm{Me}}$ ligand (L′). The $q_{\mathrm{CT}}$ values correspond to the TheoDORE charge-transfer numbers, while $deh$ denotes the root-mean-square electron–hole separation.

| State | $\lambda$ (nm) | $E_{\mathrm{exc.}}$ (eV) | $f_{\mathrm{osc.}}$ | $q_{\mathrm{CT}}$ | $d_{eh}$ (Å) | TheoDORE character contributions | | | | | |
|---|---|---|---|---|---|---|---|---|---|---|---|
| | | | | | | LMCT/L′MCT | MC | MLCT | ML′CT | LL′CT/L′LCT | LC/L′C |
| S1 | 648.8 | 1.91 | 0.036 | 0.813 | 4.426 | 3.1 % | 3.0 % | 40.3 % | 1.2 % | 36.7 % | 15.8 % |
| S2 | 533.4 | 2.32 | 0.069 | 0.876 | 4.198 | 2.0 % | 3.8 % | 57.4 % | 1.8 % | 26.4 % | 8.6 % |
| S3 | 433.1 | 2.86 | 0.007 | 0.857 | 3.754 | 1.0 % | 5.6 % | 73.4 % | 2.0 % | 9.3 % | 8.7 % |
| S4 | 412.2 | 3.01 | 0.097 | 0.859 | 4.784 | 0.4 % | 0.6 % | 46.2 % | 0.5 % | 38.7 % | 13.5 % |
| S5 | 404.4 | 3.07 | 0.001 | 0.887 | 4.147 | 1.7 % | 4.8 % | 62.5 % | 2.0 % | 22.4 % | 6.6 % |
| S6 | 394.0 | 3.15 | 0.009 | 0.879 | 4.574 | 2.5 % | 3.0 % | 46.2 % | 1.3 % | 37.9 % | 9.1 % |
| S7 | 385.1 | 3.22 | 0.000 | 0.872 | 4.577 | 0.8 % | 2.8 % | 51.5 % | 1.4 % | 33.6 % | 9.9 % |
| S8 | 382.5 | 3.24 | 0.005 | 0.882 | 4.116 | 0.5 % | 4.5 % | 68.2 % | 2.4 % | 17.0 % | 7.4 % |
| S9 | 370.7 | 3.34 | 0.326 | 0.498 | 3.765 | 1.9 % | 1.7 % | 2.9 % | 35.3 % | 9.7 % | 48.5 % |
| S10 | 342.6 | 3.62 | 0.025 | 0.908 | 4.878 | 0.2 % | 0.4 % | 48.8 % | 0.6 % | 41.1 % | 8.9 % |
| S11 | 324.4 | 3.82 | 0.204 | 0.445 | 3.431 | 2.5 % | 1.8 % | 1.6 % | 34.5 % | 5.9 % | 53.7 % |
| S12 | 321.5 | 3.86 | 0.002 | 0.894 | 4.896 | 0.4 % | 0.4 % | 46.7 % | 1.1 % | 41.3 % | 10.1 % |
| S13 | 312.6 | 3.97 | 0.034 | 0.810 | 4.946 | 0.3 % | 0.1 % | 40.0 % | 0.9 % | 39.9 % | 18.8 % |
| S14 | 295.4 | 4.20 | 0.261 | 0.737 | 4.956 | 4.6 % | 0.4 % | 6.5 % | 1.6 % | 61.0 % | 25.9 % |
| S15 | 287.2 | 4.32 | 0.261 | 0.414 | 4.431 | 3.3 % | 0.2 % | 12.1 % | 0.4 % | 25.5 % | 58.4 % |
| S16 | 283.5 | 4.37 | 0.048 | 0.736 | 4.946 | 1.3 % | 0.3 % | 26.9 % | 0.9 % | 44.6 % | 26.1 % |
| S17 | 280.0 | 4.43 | 0.028 | 0.715 | 4.709 | 0.9 % | 0.8 % | 28.2 % | 8.4 % | 34.0 % | 27.7 % |
| S18 | 277.1 | 4.47 | 0.011 | 0.600 | 4.284 | 1.5 % | 0.5 % | 42.1 % | 2.5 % | 13.9 % | 39.5 % |
| S19 | 276.3 | 4.49 | 0.021 | 0.588 | 4.343 | 1.5 % | 0.5 % | 42.6 % | 2.4 % | 12.3 % | 40.7 % |
| S20 | 273.6 | 4.53 | 0.031 | 0.366 | 4.314 | 2.0 % | 0.3 % | 5.9 % | 12.4 % | 16.2 % | 63.1 % |
| S21 | 273.2 | 4.54 | 0.036 | 0.337 | 4.382 | 2.8 % | 0.4 % | 7.6 % | 6.6 % | 16.7 % | 65.9 % |
| S22 | 272.1 | 4.56 | 0.072 | 0.463 | 4.441 | 1.9 % | 1.1 % | 10.6 % | 18.1 % | 15.7 % | 52.5 % |
| S23 | 268.5 | 4.62 | 0.034 | 0.783 | 4.806 | 0.7 % | 0.3 % | 36.4 % | 4.6 % | 36.6 % | 21.5 % |
| S24 | 267.5 | 4.64 | 0.014 | 0.824 | 4.901 | 0.7 % | 0.3 % | 41.4 % | 1.4 % | 38.8 % | 17.3 % |
| S25 | 264.6 | 4.68 | 0.009 | 0.815 | 4.575 | 0.7 % | 0.6 % | 47.8 % | 6.7 % | 26.4 % | 17.8 % |
| S26 | 263.6 | 4.70 | 0.018 | 0.820 | 4.950 | 4.0 % | 0.4 % | 10.5 % | 2.0 % | 65.4 % | 17.7 % |
| S27 | 261.2 | 4.75 | 0.016 | 0.794 | 4.645 | 3.2 % | 1.5 % | 26.0 % | 7.2 % | 43.1 % | 19.1 % |
| S28 | 261.0 | 4.75 | 0.110 | 0.752 | 3.891 | 1.7 % | 2.8 % | 11.7 % | 43.8 % | 17.9 % | 22.0 % |
| S29 | 260.7 | 4.76 | 0.149 | 0.817 | 4.454 | 2.9 % | 1.7 % | 38.8 % | 10.6 % | 29.4 % | 16.6 % |
| S30 | 260.2 | 4.76 | 0.008 | 0.832 | 4.424 | 2.1 % | 2.0 % | 53.8 % | 8.2 % | 19.0 % | 14.8 % |
